# New Insights into Ion Channels: Predicting hERG-Drug Interactions

**DOI:** 10.3390/ijms231810732

**Published:** 2022-09-14

**Authors:** Michael Fitzpatrick Wempe

**Affiliations:** 1 Department of Pharmaceutical Sciences, Skaggs School of Pharmacy and Pharmaceutical Sciences, University of Colorado—Anschutz Medical Campus, Aurora, CO 80045, USA; michael.wempe@cuanschutz.edu; Tel.: +1-303-724-8982; 2 University of Colorado Cancer Center, University of Colorado—Anschutz Medical Campus, Aurora, CO 80045, USA

**Keywords:** potassium channel, human ether-à-gogo-related gene (hERG), structure–activity relationships (SAR), ab initio calculations, drug development, long QT syndrome, *Torsades de Pointes*

## Abstract

Drug-induced long QT syndrome can be a very dangerous side effect of existing and developmental drugs. In this work, a model proposed two decades ago addressing the ion specificity of potassium channels is extended to the human ether-à-gogo gene (hERG). hERG encodes the protein that assembles into the potassium channel responsible for the delayed rectifier current in ventricular cardiac myocytes that is often targeted by drugs associated with QT prolongation. The predictive value of this model can guide a rational drug design decision early in the drug development process and enhance NCE (New Chemical Entity) retention. Small molecule drugs containing a nitrogen that can be protonated to afford a formal +1 charge can interact with hERG to prevent the repolarization of outward rectifier currents. Low-level ab initio calculations are employed to generate electronic features of the drug molecules that are known to interact with hERG. These calculations were employed to generate structure–activity relationships (SAR) that predict whether a small molecule drug containing a protonated nitrogen has the potential to interact with and inhibit the activity of the hERG potassium channels of the heart. The model of the mechanism underlying the ion specificity of potassium channels offers predictive value toward optimizing drug design and, therefore, minimizes the effort and expense invested in compounds with the potential for life-threatening inhibitory activity of the hERG potassium channel.

## 1. Introduction

While recent studies on potassium channels have expanded our understanding of their structure and function, there were three physiochemical phenomena whose underlying mechanism remained to be explained: (1) the selectivity for K^+^ despite higher extracellular levels of Na^+^, (2) pore selectivity being enforced despite the larger size of the potassium ion compared to the sodium ion, and (3) the TEA (tetraethyl ammonium cation) being an effective inhibitor of potassium channels, whereas the smaller TMA (tetramethyl ammonium cation) has little to no effect despite the intuitive notion that its smaller size might permit deeper penetration in the pore. One explanation for these phenomena was proposed in a previously presented model [1].

**Figure 1:**
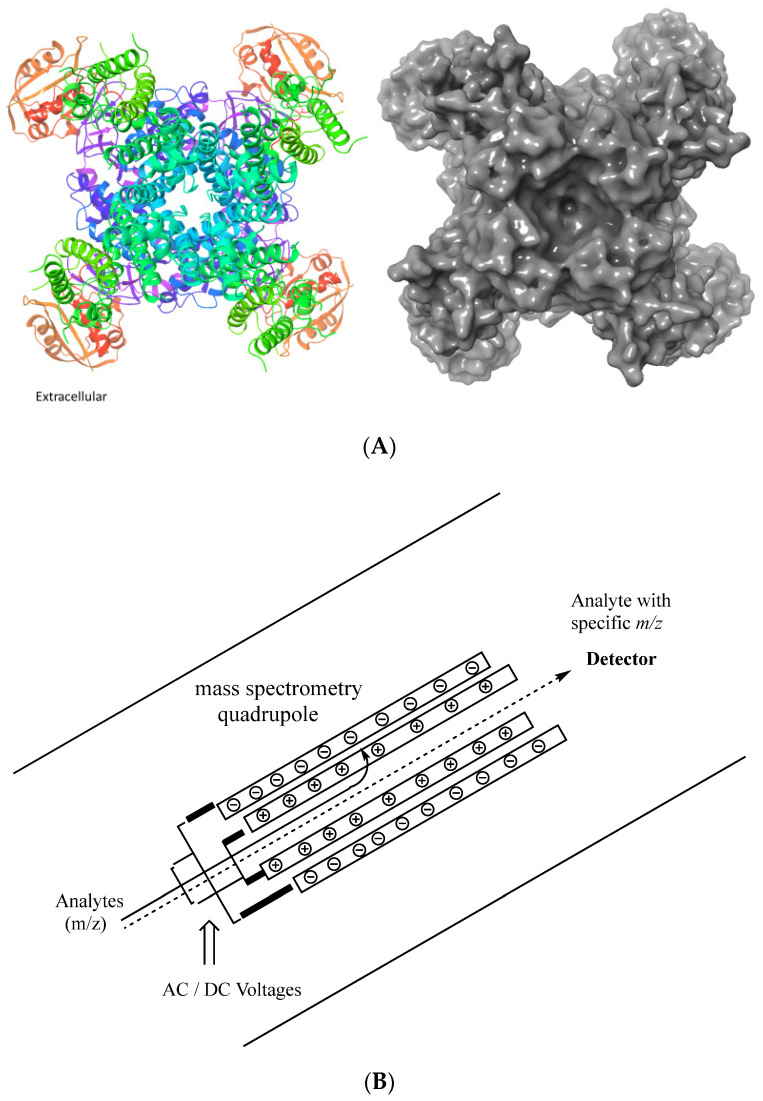
(**A**) Representative depiction of an extracellular view of a voltage-gated potassium channel. Ribbon representation of the hERG channel. The image was generated using Schrӧdinger Maesto and importing the PDB: 5VA1 3.70 Å structure [2], including the biological unit. The ribbons are colored by residue position using the default palette in Schrӧdinger Maestro. (**B**) Representative sketch of a mass spectrometry quadrupole.

**Figure 2:**
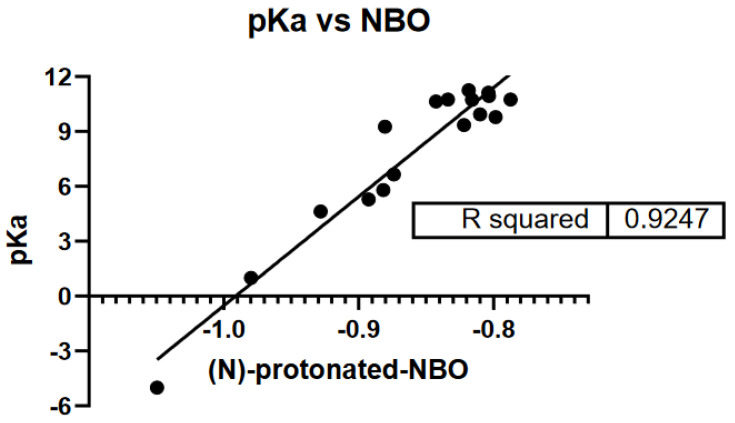
Protonated amines atomic charge, pKa vs. HF/321G NBO data.

The model addresses the ion specificity of potassium channels by noting that an *m/z* relationship exists for quaternary ammonium (QA) ions. An analogy to quadrupole mass spectrometry (QMS) was proposed [1]. Potassium channels have four alpha-subunits (Figure 1A; [2]) with a membrane potential. In QMS, there are four parallel rods with two opposite rods having an applied potential of [U+Vcos(wt)] and the other two are -[U+Vcos(wt)], where Vcos(wt) is an ac voltage while U is a dc voltage (Figure 1B) [3,4]. Applied voltages influence the ion trajectory traversing down the channel. Thus, in QMS, the ion beam is directed in a longitudinal direction and resides between four parallel opposite rods with their phases shifted 180 degrees from one another; they are superimposed by a high-frequency field. In the case of a pre-defined current, only particles of a particular *m*/*z* will reach the outer slot. The gating kinetics regarding potassium channels are multifaceted [5,6,7]; the fact that it is generally accepted that all voltage-gated potassium channels have essentially the same pore construction allows one to extend the QA ion model proposed previously [1] to predict drug-hERG inhibition. One investigates the charge on the protonated nitrogen, the alkyl environment surrounding the protonated amine, and then the aromatic skeleton found within the drug substance.

### 1.1. hERG and Potassium Channel History

In 1994, Warmke and Ganetzky [8] recognized the novel human cDNA, now known as the human ether-à-gogo-related gene (hERG). Located in human ventricles and human atria, the hERG encodes the potassium channel responsible for the delayed rectifier current Ikr [9,10]. The repolarization of cardiac myocytes can be triggered by the activation of outward potassium ion currents and involves the delayed rectifier current (IK). The delayed rectifier current contains two components, Ikr and Iks. Many drugs associated with QT prolongation have been shown to block Ikr and/or the cloned hERG channel. Therefore, understanding the molecular and physicochemical mechanisms involved in voltage-gated potassium channels is important.

Potassium channels are water-solvated proteins that allow certain ions to pass through the pore [11] and transport across the lipid bilayer membrane. All potassium channels have nearly the same pore construction as shown by molecular cloning and mutagenesis studies. Biophysical data support the notion that the potassium channel pore region possesses multiple binding sites, has a high affinity for K^+^, and acts as a selectivity filter [12]. The four α-subunits form a cone that cradles the selectivity filter; therefore, K^+^ channels are long, narrow pore channels that produce an ion flux. Potassium channels may be thousands of times more permeable to K^+^ than to Na^+^. Known as the ‘potassium channel signature sequence’, potassium channels contain critical amino acids, and the mutation of these amino acids can disrupt the preference for K^+^ over Na^+^ [12].

### 1.2. Long QT Syndrome

Drug-induced long QT syndrome can be a dangerous side effect of certain prescription drugs. It is commonly accepted that different drugs may prolong cardiac repolarization by blocking one or more types of voltage-gated K^+^ channels. As a result, drugs that create the prolongation of the QT interval on the electrocardiogram (EKG) can lead to the life-threatening ventricular arrhythmia known as *Torsades de Pointes* [13]. The fact that all of the known compounds (>95%) which produce these physiological effects contain an amine functionality and the fact that it is generally accepted that all voltage-gated potassium channels have essentially the same pore construction (i.e., four α-subunits form a cone that cradles the selectivity filter) allow one to extend the QA ion model proposed in 2001 [1] to predict the potential of a drug to inhibit hERG and, therefore, the prospect of drug-induced prolonged QT syndrome.

### 1.3. Potassium Channel Research and Development

In the past two decades, a number of research groups have addressed issues of K^+^ channel specificity for ions and inhibitors. Several studies employing a variety of approaches offer insight into hERG and its interactions with drugs and ions. In 2012, Vilums et al. reported an interesting hERG SAR on the inhibitor known as E-4031 [14]. Other researchers performed hERG SAR studies, for example, on dofetilide derivatives [15]. Moreover, in 2013, Louvel et al. [16] reported an SAR around the Clofilium template. Other groups investigated hERG inhibition in a large diverse compound library by performing an automated patch-clamp assay [17]. In addition, Negami et al. [18] computed hERG-drug binding free energies. Recently, a group combined multi-dimensional molecular fingerprints to predict hERG [19]. Butler et al. provided a nice review of the hERG structure [20], providing information regarding mutagenesis studies and channel gating. The current work provides an alternative way of looking at the hERG-drug interactions by looking at the atomic charges on the atoms within molecules, specifically protonated amines. Our hope is that the current methods will help medicinal chemists and preclinical safety scientists with an alternative way to look at hERG-drug interactions early in the New Chemical Entity (NCR) developmental process.

## 2. Results and Discussion

### 2.1. Fundamental Concepts: SAR, Drift Speed, and Effective Charge

Models and experiments that assess biological activity as a function of chemical structure are called ‘Structure-Activity Relationships’ (SAR) [21]. SAR models can provide qualitative and/or quantitative predictions that help decipher critical chemical motifs, chemical mechanism, and chemical trends [22]. Our model proposes that potassium channels segregate ions in a manner analogous to a quadrupole mass spectrometer governed by an *m/z* relationship. Thus, it is critical to define the effective charge on the protonated amine nitrogen within the drug molecule. An effective charge on the quaternary amine is determined by the effect of the molecular environment (via through-bond, electronic, inductive, and through-space interactions) and the extent of the solvation of the molecule.

In the case of ions (i.e., protonated amines in small drug molecules, versus small ions), when an ion moves through a solvent, such as water, it experiences an impeding frictional force proportional to its speed. If one accepts that the Stokes’ relationship applies at the molecular scale, then a moving aqueous ion will reach a terminal speed known as the ‘drift speed’. The drift speed is achieved when the accelerating force of the ion is balanced by the viscous drag. Hence, it is the drift speed that governs the rate at which a charge can be transported. Molar conductivity decreases with increasing ion size, but this is not true for exceedingly small ions. Small ions, such as Li^+^ and Na^+^, generate a stronger electric field than larger ions, such as TEA. Because Li^+^ and Na^+^ drag water molecules as they migrate, these small cations are solvated to a greater extent and generate a larger hydrodynamic radius. Therefore, when one computes the estimated effective hydrodynamic radii, a Li^+^ ion drags approximately three water molecules, while a Na^+^ ion drags one water molecule, and potassium or larger ions, unless they have hydrogen bonding capacity (FON Bonds), do not readily drag along water molecules.

The presented model is purposely based on a low level of computational theory performing gas-phase calculations; higher levels of computational theory are not needed to unveil trends. The results obtained using Hartree–Fock (HF/321G) and the Chem3D property server are summarized in Table S1 and support the notion that the Connolly molecular area and the Connolly solvent-excluded volume order for small ions has the following trend: 3H_2_O·Li^+^ > H_2_O·Na^+^ > Rb^+^ > K^+^ > H_2_O > Na^+^ > Ca^+2^ > Mg^+2^ > Li^+^. Hence, the hydrated sodium ion is larger in size than a non-hydrated potassium ion—computed atomic masses (Na^+^ hydrate 41.0 amu; versus K^+^ 39.1 amu).

In the case of a multi-electron system, such as Na^+^ and K^+^, each electron is attracted to the nucleus and experiences a Coulombic repulsion by the other electrons. The electron density between the nucleus and the electron of interest will reduce the nuclear charge acting on that electron. The net positive charge attracting the electron is known as the ‘effective nuclear charge’, Z_eff_. ‘Z_eff_ = Z − X’ where ‘Z’ is the number of protons in the nucleus and ‘X’ is the number of electrons between the nucleus and the electron of interest. Hence, an outer electron experiences a shielded nuclear charge but does not oppose the full Coulombic attraction of the nucleus. The effective charge is simply a way of expressing the net outcome of the nuclear attraction and the electronic repulsions in terms of a single equivalent charge at the center of the atom [23,24]. On the basis that the shielding of a nuclear charge occurs in a multi-electron system, alkyl groups covalently attached to the nitrogen atom in a quaternary ammonium ion will shield the formal +1 charge [1]. We approached the problem by using different methods to computationally compute effective charges on the atoms within a molecule where the amine is protonated.

### 2.2. Computing Atomic Charges

Here, we asked whether the concepts proposed previously [1] can be extended to the drug-hERG SAR. Many drugs and New Chemical Entities (NCEs) contain at least one amine functional group. Amines (primary, secondary, and tertiary), having a lone pair of electrons, can be protonated to afford a quaternary ammonium cation. Thus, an equilibrium between protonated and non-protonated forms exists at the pH values characteristic for each molecule. As illustrated experimentally and theoretically, Table S2, the substituent(s) attached to the amine inherently influences the observed pKa.

There are a variety of ways to computationally generate atomic charges [25]. The three different methods that were computed in this manuscript include: (1) the Mulliken population analysis that will partition the total +1 molecular charge amongst the atoms within the molecule; (2) the natural population analysis (NBO) which uses core electrons, valence electrons, and electrons located in diffuse functions to partition the charge on the nitrogen; and (3) the Merz–Kollman–Singh (MKS) method which assigns point charges to fit the computed electrostatic potential to points on concentric spheres around each atom. Out of the three methods, the Mulliken and NBO were found to be more informative than the MKS. Regarding the Mulliken Population Analysis, Mulliken charges provide a way to estimate partial atomic charges from calculations carried out by computational chemistry via a linear combination of atomic orbitals (LCAO), a molecular orbital method routinely used as variables in linear regression, whereas the NBO analysis is based on a method that optimally transforms a given wave function into a localized form which corresponds to Lewis structure pictures where one has lone pairs and chemical bonds.

Because amines form weaker hydrogen bonds compared to alcohols, their boiling points, in general, are lower and their solubilities in water are less. Tertiary amines are least solvated, while primary amines are the most solvated. In addition, as represented in Figure 2, there is a correlation (R squared 0.92) between the experimental pKa and the computational values computed on the protonated nitrogen atom (NBO); the amine basicity is derived from a combination of inductive, steric, and solvation effects.

### 2.3. Generating a Modality That Predicts Drug-hERG Interaction: How Rational Drug Design Can Avoid This Pitfall

Researchers have used a number of different experimental techniques to evaluate drug-hERG interactions in vitro. Gonzalez et al. [26] reviewed cell-based assays used to analyze the properties of ion channels, including (i) electrophysiology (patch-clamp) binding assays; (ii) radioactive flux assays; (iii) redistribution membrane-potential dyes; and (iv) FRET-based voltage sensors. A variety of drugs that may prolong the QT interval and/or induce *Torsades de Pointes* have been summarized in Table 1 (compounds **1**–**54**); these compounds represent the ‘learning compound set’. Their chemical structures are presented in the Supplementary Materials section, Figure S1. Experimentally determined IC_50_ values for compounds (Table 2) were collected from the literature. The IC_50_ values are derived from hERG-transfected HEK, COS-7, CHO or neuroblast cells, and hERG K^+^ channels expressed in *Xenopus laevis* oocytes or zebrafish model [27,28,29,30,31,32,33,34,35,36,37,38,39,40,41,42,43,44,45,46,47,48,49,50,51,52,53,54,55].

**Figure 3:**
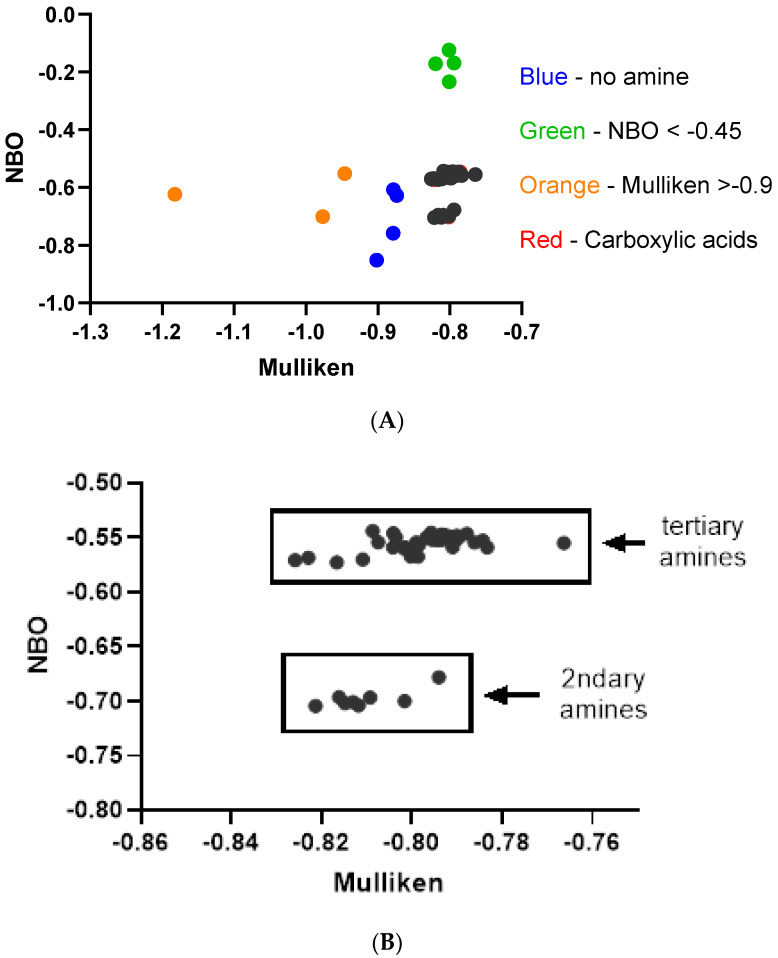
(**A**): Table 1 Compounds HF/321G NBO vs. Mulliken Charge. (**B**): NBO vs. Mulliken Charge, secondary and tertiary amines; Table 1 protonated compounds after questions 1–5.

**Scheme 1:**
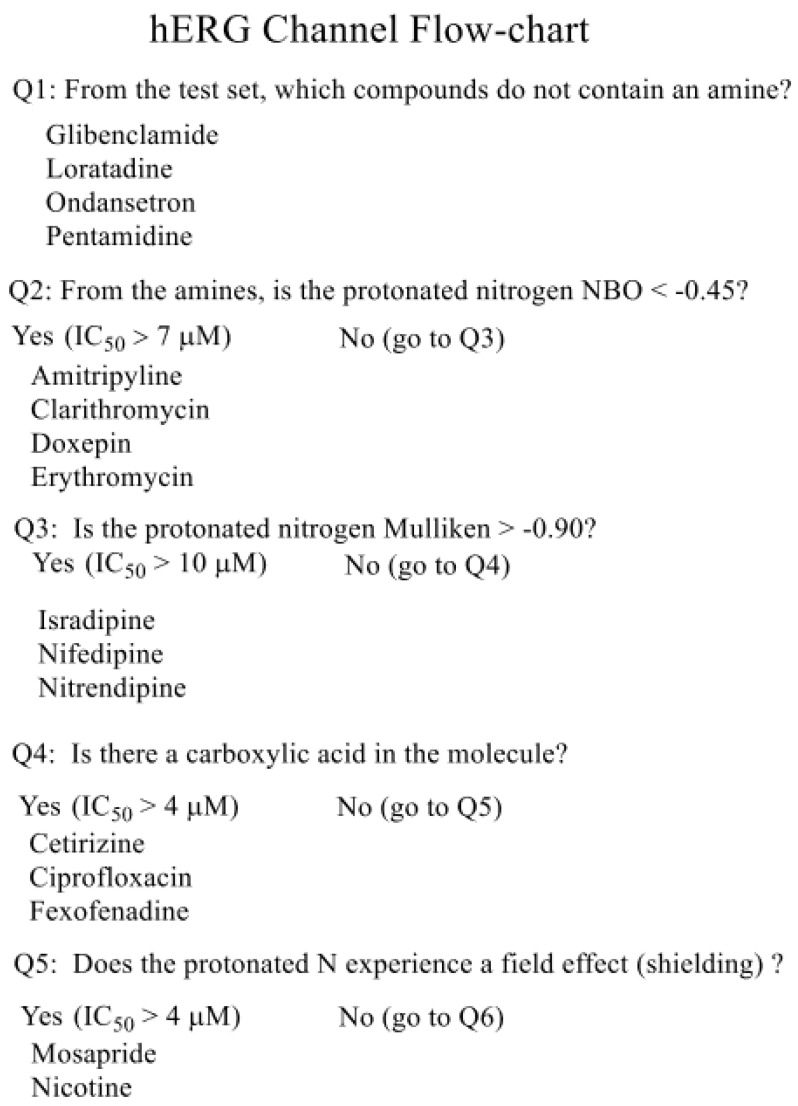

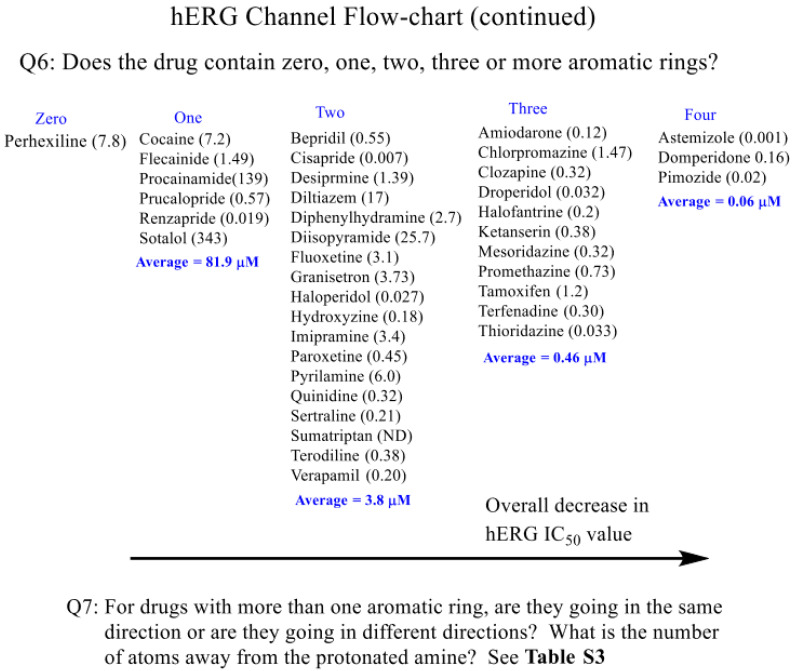
hERG channel prediction flow chart for first compound set.

The compounds were computationally evaluated, and their data were also summarized in Table 2. The NBO values versus Mulliken were plotted to provide the electronic patterns (Figure 3A) for these compounds. As summarized in the flow chart (Scheme 1), a series of initial questions were asked to eliminate the compounds without amines and poor interactors. Question 1: Glibenclamide (**23**; 74 μM), loratadine (**31**; 0.17 μM), ondansetron (**37**; 0.81 μM), and pentamidine (**39**; 5–8 μM), **blue** points, do not contain an amine and are, therefore, not subjects for this scheme. Question 2: Amitripyline (**2**; 10 μM), clarithromycin (**9**; 720 μM), doxepin (**17**; 7 μM), and erythromycin (**19**; >10 μM), Figure 3A, **green** points > 7.0 μM, all exhibit high IC_50_ values and have an NBO < −0.45. Question 3: Isradipine (**29**; >10 μM), nifedipine (**35**; >50 μM), and nitrendipine (**36**; >10 μM), Figure 3A, **orange** points, exhibit low hERG affinity and possess a protonated nitrogen Mulliken charge > −0.90. Question 4: Compounds possessing a carboxylic acid are all low-affinity compounds. These compounds, Cetirizine (**5**; >30 μM), ciprofloxacin (**7**; 966 μM), and fexofenadine (**20**; 214 μM), are designated by red points but are embedded in the remaining data points in black, Figure 3A. In Figure 3B, the low-affinity interactors identified by questions 1–4 (colored points from Figure 3A) have been removed. This produces a plot with two distinct electronic patterns largely containing hERG inhibitors with low IC_50_ values. One group is composed of secondary amines (HF/321G NBO −0.698 ± 0.009), while the other group is tertiary amines (HF/321G NBO −0.555 ± 0.008). It is in these two electronic regions where potent hERG inhibitors are found.

Within these two groups there are several outliers with a low affinity for hERG, Figure S2. Some outliers are identified by question 5: Does the protonated nitrogen experience a strong field effect? Nicotine (**34**; 245 μM) and mosapride (**33**; 4.8 μM) have statistically higher IC_50_ values due to the strong field effect (shielding) [1]. Procainamide (**42**; 139 μM) and sotalol (**49**; 78 μM) also exhibit high IC_50_ values. For sotalol, the increase in the IC_50_ value appears to be due to intramolecular hydrogen bonding via the N-CH_2_-CHOH-Ph functionality. The high IC_50_ of procainamide may be attributable to the resonance structure of the aromatic amine with the amide and relative distance to the protonated alkyl amine. More selective SAR data would help evaluate this speculation as certain potent, low IC_50_ compounds, such as cisapride, prucalopride, and renzapride, all contain Ar-NH_2_ and alkyl amines.

As well as the alkyl environment surrounding the nitrogen, the presence of aromatic rings also has a major influence on the IC_50_ values. For secondary amines containing two aromatic rings, the IC_50_ (μM) trend was fluoxetine (**22**; 3.1 μM) > desipramine (**12**; 1.39 μM) > paroxetine (**38**; 0.45 μM) > terodiline (**51**; 0.375 μM) > sertraline (**48**; 0.210 μM)—summarized in Table S3. Attempts were conducted to look at other indicators, such as the molecular weight (267–415 amu), cLogP values, and number of heteroatoms. Irrespective of these parameters, the number of aromatic rings and the distance from the alkyl amine, whether or not the aromatic rings are in the same direction or opposite directions, and the alkyl amine environment are the major factors dictating the in vitro IC_50_ values.

In general, as inferred from the data summarized in Scheme 1 Q6, a general trend of more potent, lower hERG IC_50_ values occurs as one goes from two to three to four aromatic rings, respectively. In general, secondary amines have hERG IC_50_ values greater than tertiary amines. Regarding the molecules containing two aromatic rings but with tertiary amines (Table S3), the IC_50_ trend ranged from 0.0067 to 25.7 μM. The most potent ones having the aromatic groups being in an opposite direction and separated by 7–10 bond distances between the aromatic rings. Thus, in general, molecules containing a secondary amine, with two aromatic rings separated by different distances, have higher IC_50_ values as compared to molecules that contain a tertiary amine with a similar aromatic connectivity/framework. The compounds with three aromatic rings and possessing tertiary amines provided the following trend: chlorpromazine (**6**; 1.47 μM) > tamoxifen (**50**; 1.2 μM) > promethazine (**43**; 0.73 μM) > ketanserin (**30**; 0.38 μM) > mesoridazine (**32**; 0.32 μM) = clozapine (**10**; 0.32 μM) > terfenadine (**52**; 0.21 μM) > halofantrine (**25**; 0.20 μM) > amiodarone (**1**; 0.118 μM) > thioridazine (**53**; 0.036 μM) > droperidol (**18**; 0.032 μM) – (range 0.032–1.47 μM). There is a general trend of more potent hERG inhibitors with three aromatic rings as compared to two aromatic rings.

Finally, the three examples of four aromatic rings and tertiary amines were domperidone (**16**; 0.16 μM), pimozide (**41**; 0.018 μM), and astemizole (**3**; 0.001 μM), all potent (< 0.1 μM) hERG inhibitors with aromatic groups in opposite directions and 7–10 bond distances from the aromatics. For example, the distance of the aromatic ring(s) from the amine nitrogen alkyl environment is as important as the alkyl environment surrounding the amine.

The current proposal does not explain the higher experimental IC_50_ values for diltiazem (**13**; 17 μM) or disopyramide (**15**; 25.7 μM)—Figure S3—as one might predict these to be more potent inhibitors. For diltiazem, the increased IC_50_ may be a function of the number and location of heteroatoms providing repulsive interactions, resulting in a high IC_50_, but a systematic SAR is needed. While not a perfect comparison, if one compares disopyramide (**15**; 25.7 μM) to diphenylhydramine (**14**; 2.7 μΜ), the distance of the 3,3-diaromatic from the protonated amine, as compared to the distancing by 4,4-di-aromatic, gives diphenylhydramine with an IC_50_ of 2.7 μM. There is about a 10-fold reduction in the IC_50_ from distancing by one methylene unit to afford a 4,4-aromatic connectivity. This provides some SAR information. Alternatively, the amide in disopyramide may be contributing intramolecular hydrogen bonding properties and causing the IC_50_ to be greater than what one would predict from the computational data. Thus, while looking to make hERG inhibition predictions, one needs to perform the computational analysis, ask the questions in the flow chart, and assess the alkyl environment (secondary versus tertiary).

As summarized in Scheme 1, Q6: from the list of drugs that may prolong QT Table 1, the remaining drugs are grouped in terms of zero, one, two, three, and four aromatic rings. While a generalization, the amine as a secondary or a tertiary amine plays a factor, and the number of aromatic groups disseminating from the amine functionality is also a factor. For example, going from left to right (Scheme 1, Q6; zero to four aromatic rings) has a general average trend of more potent hERG inhibitors in vitro (higher to lower IC_50_ values, respectively). For all groups containing at least one aromatic ring, it is important to note that all contained at least one potent (<0.10 μM) inhibitor, e.g., group one, renzapride (**47**; 0.019 μM); group two, cisapride (**8**; 0.007 μM); group three, droperidol (**18**; 0.032 μM); and group four, astemizole (**3**; 0.001 μM).

Next, the alkyl environment around the amine was assessed. The compounds with two or more aromatic rings in the compound trigger the question: ‘are the aromatic rings going in the same or different directions, and what connectivity may they exhibit?’ (Q7). These considerations are summarized in Table S3 for the compounds that have two, three, and four aromatic groups, with their *N*-alkyl environment, relative distance from the amine, connectivity, and whether or not the groups are going in the same direction or opposite directions described. These again help to illustrate an important point; in cases where the aromatic rings are in opposite directions and within 7–10 bonds distance or so between the aromatic rings, all have very low IC_50_ values (<0.1 μM), such as astemizole (**3**; 0.001 μM); pimozide (**41**; 0.018 μM); cisapride (**8**; 0.007 μM); haloperidol (**26**; 0.027 μM); and droperidol, (**18**; 0.032 μM). If the aromatic rings are in the same direction, they have higher IC_50_ ranges (0.1–1.0, 1.0–2.0, and 2.0–4.0 μM), but more experimental examples and a systematic SAR on a handful of chemical templates are needed.

### 2.4. Examples Illustrating the QA Ion Model

#### 2.4.1. Example One: Terfenadine versus Fexofenadine

Terfenadine is a classic example of where the major route of metabolism is catalyzed by CYP (cytochrome P450 2D6 and 3A4)-mediated oxidation [56] to give fexofenadine and that the co-administration of a potent CYP3A4 inhibitor with terfenadine, such as ketoconazole, can result in elevated terfenadine plasma concentrations and lead to life-threatening results [57,58]. Terfenadine has a reported IC_50_ for hERG of 31 to 300 nM (Table 2). The phase I metabolic biotransformation of terfendadine to the corresponding carboxylic acid (fexofenadine) is predicted by the QA ion model to greatly increase the IC_50_ (Scheme 1, question 4). Indeed, the reported IC_50_ for fexofenadine is more than 100-fold higher than for terfenadine [29]. As illustrated in Figure S4, the protonated terfenadine nitrogen is assessable to the channel pore, whereas in the case of fexofenadine, the carboxylic acid anion becomes attracted to the ammonium ion, Coulombic attraction interactions and sterically blocks interactions with the hERG pore region. Hence, giving rise to a much higher hERG IC_50_. Thus, a strategy is to impede the accessibility of the amine to hERG.

#### 2.4.2. Example Two: Cisapride, Mosapride, Prucalopride versus Renzapride

Using four different serotonin 5-hydroxytryptamine (5-HT4) receptor agonists (cisapride, mosapride, prucalopride, and renzapride), González et al. [27] described the investigations with hERG-transfected COS-7 cells utilizing the patch-clamp procedure. In summary, they were able to rank order the 5-HT4 potency to the hERG blockade: mosapride (**33**; 4800 nM) >> prucalopride (**44**; 570 nM) > renzapride (**47**; 180 nM) >> cisapride (**8**; 6.5–24 nM; [59,60,61]); mosapride did not produce a significant response in the recombinant hERG current. At first glance, when one compares the protonated forms of these four compounds, Figure S5, it may not be inherently obvious that mosapride is chemically distinct. However, by considering that a protonated amine can be influenced by field effects [1], then these 5-HT4 trends can be explained. The *p*-fluorobenzyl functionality in the protonated mosapride results in a strong field effect which alters the effective charge (Z_eff_) on the nitrogen atom; a decrease in the effective formal charge (*Z*) results in a lower affinity, a higher IC_50_, toward the hERG channel.

### 2.5. Testing the Ammonium Ion Model

The development of this proposed way to evaluate hERG interactions should allow medicinal chemists and ADME/DMPK researchers/scientists the opportunity to work together and make rational decisions about potential hERG liabilities prior to conducting extensive synthesis and animal testing. The ability to chemically dial-out and/or tone-down potential life-threatening drug-hERG interactions in vivo during NCE (New Chemical Entity) optimization should be of value to researchers and pharmaceutical companies.

The insights gained from the learning set of the compounds allows one to make predictions regarding a test set of drugs and/or metabolites; Table 3, (Compound Set; **55**–**91**); the chemical structures are in Figure S6. First, using the question sequence Scheme S1, predictions were made and incorporated into Table 4. The predictions were made based upon the sequence of questions and comparisons to the examples in the learning set, i.e., overall reflecting the number of aromatic rings, connectivity, alkyl environment of the protonated amine, etc. Next, the literature data were sought, tabulated, and used to compare to the predictive ranges. All of the compounds in the test set contain an amine (Scheme S2), and only one compound (**57**; clozapine-*N*-oxide) had an NBO < −0.45; therefore, the prediction is > 7.0 μM with a reported experimental value of 133 μM.

Q3: Nicarpidine (**80**) in the test set exhibits a protonable nitrogen with a Mulliken value -0.90 and thus has an estimate of >10 μM. Proceeding to question 4, the following compounds of the test set have a carboxylic acid moiety and therefore are predicted to have an IC_50_ greater than 6.0 μM: bilastine (**56**; 6–17 μM); carebastine (**61**; estimated at > 6.0 μM); 2-carboxy-methyl-olanzapine (**65**; estimated at >6.0 μM); gatifloxacin (**73**; 50 μM); levofloxacin (**75**; 915 μM); and sparfloxacin (**89**; 18 μM).

Compounds **56** and **61** are analogous to the ‘example one’ scenario previously discussed for terfenadine versus fexofenadine, where intramolecular attraction impedes the hERG interaction via intramolecular steric hindrance and Coulombic attraction, resulting in a higher IC_50_. In regard to the remaining compounds in the test set, the number of aromatic rings and relative connectivity (structures) are summarized in Scheme S1 and Table S4. A general trend of increased aromatic rings is associated with a lower hERG IC_50_, a trend seen with the learning set. Furthermore, the general trend of the aromatic rings being in different directions gives rise to lower IC_50_ values also holds true with the test set.

From the summary in Table S4, the following compounds from the test set were predicted to have IC_50_ values < 0.1 μM: O-desmethyl-astemizole (**59**; 0.01 μM), dofetilide (**67**; 0.007 μM), E-4031 (**71**; 0.0268 μM), MK-499 (**78**; 0.032 μM), lidoflazine (**74**; 0.037 μM), sertindole (**87**; 0.024 μM), and norastemizole (**81**; 0.028 μM). Dofetilide, E-4031, and MK-499 were all potent inhibitors. An interesting observation is that these last three compounds all have a sulfonamide functional group Ar-NHSO_2_Me extending away from the amine functionality, contributing to their inhibition potency. The compounds with hERG IC_50_ values predictions between 0.1 and 1.0 μM (group one) include risperidone (**85**; 0.14 μM), ziprasidone (**91**; 0.15 μM), DSP-6952 (**69**; 0.271 μM), azimilide (**55**; 0.58 μM), prenylamine (**82**; 0.59 μM), 2-hydroxy-olanzapine (**64**; 0.23 μM), olanzapine (**62**; 0.231 μM), ebastine (**60**; 0.30 μM), and clozapine (**10**; 0.32 μM).

Group two (1.0–4.0 μM) includes: Sildenafil (**88**; 3.3 μM), 9-hydroxy-risperidone (**86**; 1.3 μM), mibefradil (**76**; 1.43 μM), endoxifen (**72**; 1.6 μM), propafenone (**83**; 2.0 μM), and CJ-033466 (**66**; 2.6 μM); and those at or > 4.0 μM are desmethyl clozapine (**58**; 4.49 μM), dolasetron (**68**; 5.95 μM), MDL 74,156 (**79**; 12.1 μM), chlorphenamine (**70**; 13 μM), desmethylolanzapine (**63**; 14.2 μM), and mexiletine (**77**; >10 μM). Regarding propafenone **83**, one might predict it to be a lower IC_50_ value, but this example is similar to sotalol **49** where the intramolecular interactions with the alcohol functionality led to a higher IC_50_ value; so, the removal of the alcohol functionality should give rise to a more potent hERG inhibitor. In general, summarized in Table 4, the range estimates provide good predictability and can be a mechanism for go/no-go decision making during the drug development process and to guide the chemical modification of the drug substance.

**Figure 4:**
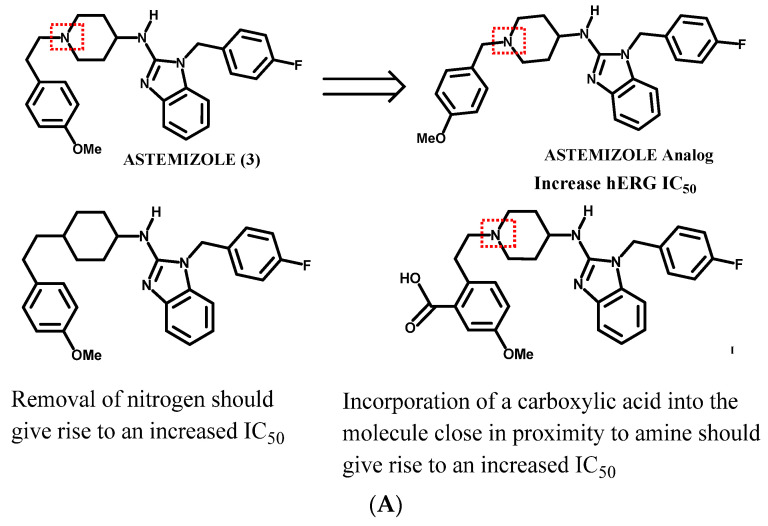

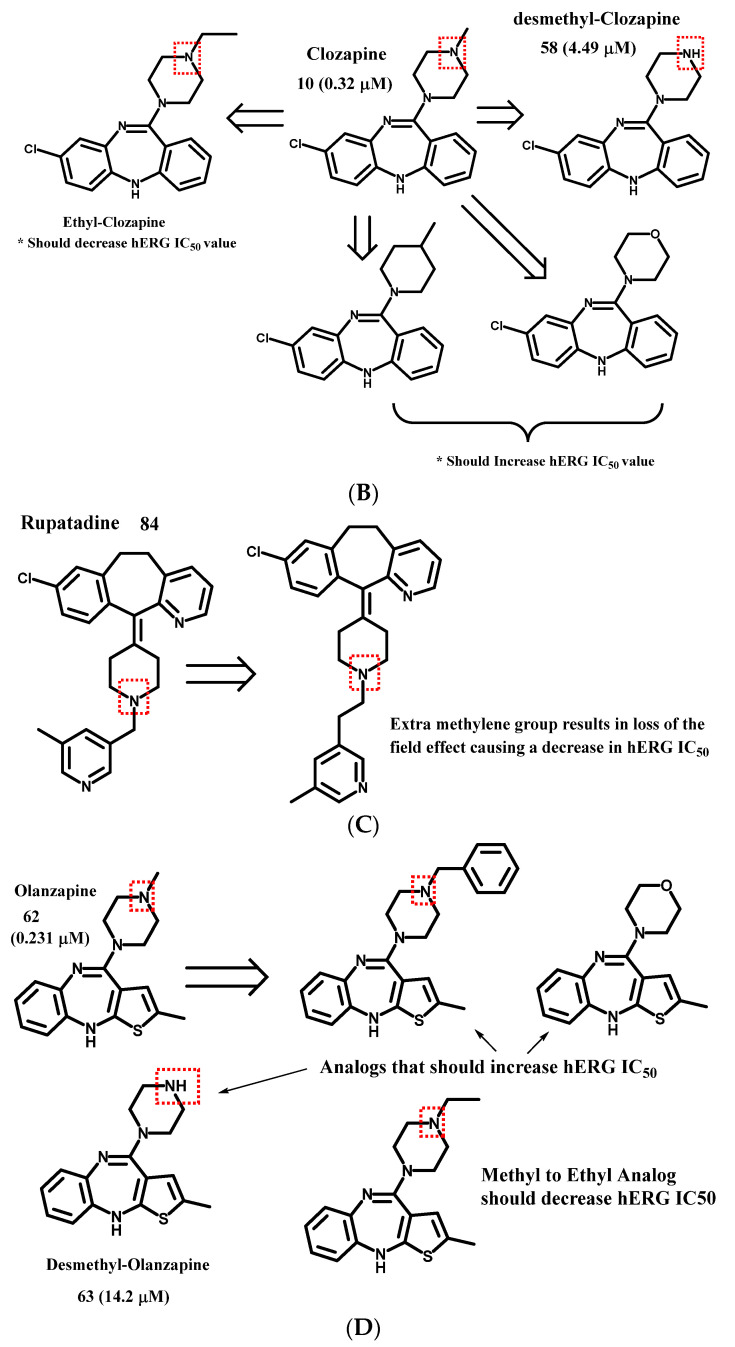
Examples of hERG interaction trend predictions, (**A**) Astemizole template. Removal of the -CH2- should give rise to a molecule whose protonated nitrogen undergoes a ‘field effect’ and thus should give rise to a molecule that has a higher hERG IC_50_. (**B**) Clozapine template. (**C**) Rupatadine template. (**D**) Olanzapine template.

The current work adds value to the preclinical safety/medicinal chemistry interface, as one can make rational drug design suggestions about how to modify an NCE that possesses serious hERG liabilities to relieve the potential safety concern that accompanies an hERG inhibitor. At the same time such modifications must strike a balance; one does not want to compromise the desired potency and other ADME properties of the NCE while optimizing the safety profile. Consequently, one could take different strategies, as proposed per the examples presented in Figure 4A, where astemizole (**3**; 0.001 μM)—a very potent hERG inhibitor—is predicted to have a statistically significant shift in the IC_50_ by removing a methylene -CH_2_- and thus affording a ‘field effect’ which should result in an increased IC_50_ (better safety profile) as compared to the parent astemizole. A similar safety improvement might be achieved by substituting the protonated nitrogen with a carbon atom. This modification would help to distinguish the importance of the amine functionality from the contributions of the aromatic skeleton. Lastly, one could incorporate a carboxylic acid into the molecule which should also give an increased hERG IC_50_.

As another example, clozapine (Figure 4B; 0.32 μM, **10**) has the potential to be a safety concern. The removal of the methyl on the methyl-piperazine (**58;** experimentally confirmed, 4.49 μM), conversion of the nitrogen to a carbon, or replacement of the methylamine with a morpholine analog are predicted to have higher hERG IC_50_ values as compared to the parent drug. On the other hand, the conversion of the methyl-piperazine to an ethyl-piperazine is predicted to result in an analog with a more potent (lower) IC_50_ value, and a worse hERG safety profile. With regard to the example (Figure 4C) rupatadine **84**, a drug that benefits from a ‘field effect’, the addition of a methylene group would disrupt this field effect and thus be predicted to have a significantly lower hERG IC_50_ value (worse safety profile). Whereas Olanzapine (Figure 4D; **62,** 0.231 μM), as an example, may be improved by converting to a benzyl functionality which should give an increased IC_50_ value due to the ‘field effect’ or the conversion of the methyl-piperazine to a morpholine functionality with an improved, higher hERG IC_50_ value. One could also remove the methyl group to afford a secondary amine which would produce a higher IC_50_ value compared to the parent compound. This projection is confirmed by the experimental data; the measured IC_50_ for compound **63** is 14.2 uM.

## 3. Methods

The computational methodology used herein was based upon calculations performed with Gaussian^®^ (Gaussian, Inc.; Pittsburgh, PA, USA) [61]. All chemical structures were drawn using CS Chem-Draw Ultra^®^ and copied into CS Chem3D Ultra^®^ (Perkin Elmer; Waltham, MA, USA). A molecular mechanics (MM) minimization was perfrmed with a root-mean-square (RMS) gradient of 0.01. Afterward, semi-empirical Austin-model (AM1) calculations were performed, and then low level ab initio (HF/3-21G) molecular orbital (MO) calculations were computed. Atomic charges were computed, and calculations were conducted using an HP Pavilion g7 Notebook PC. Data were tabulated from the output files and values plotted using GraphPad Prism 9.3.1 (1992-2021 GraphPad Software, LLC; San Diego CA, USA). Using known hERG literature in vitro data, comparisons to the computed theoretical data are presented.

## 4. Conclusions

The current work provides insights into how one may recognize the potential hERG liability in a lead molecule (NCE) that contains an alkyl-amine functionality and how rational chemical modifications may lead to compounds with a better hERG preclinical safety profile. The array of drugs with diverse chemical structures which have been investigated in this work supports the notion that there are multiple drug binding sites in the hERG pore, which adds complexity to the overall picture. More fundamental chemical information is needed to fully appreciate the overall hERG SAR. What has been presented herein is one approach. Two critical aspects need to be carefully and methodically studied: i) Carefully understanding the changes in the alkyl environment surrounding the amine while holding the aromatic framework constant; the m/z relationship is believed to be a function of the mass within four bond distances from the amine (not the entire mass of the compound). ii) Carefully probing the interactions of modified aromatic rings (i.e., electron withdrawing groups and electron donating groups, stereochemistry, etc.) More direct comparisons are needed, and future work in our lab will focus on the chemical synthesis and testing of analogs to further probe the hERG SAR. Lastly, when considering safety, it is also important to understand the extent and the chemical nature of the metabolism of the parent drug, which CYP’s and/or drug transporters are involved, etc. Other aspects are important, such as the route of the administered dose, frequency of dose, C_max_, T_max_, T_1/2_, protein binding, route of elimination, drug transporter interactions, DDI (drug–drug interactions), and pharmacodynamics (PD). Hopefully, insights from this manuscript and future work will translate into improved rational drug design for medicinal/ADME/DMPK scientists with hERG safety in mind during the NCE optimization process.

## Figures and Tables

**Table 1:** Compounds that may prolong QT and/or induce *Torsades de Pointes*.

	Drug	Classification	Drug		Classification
**1**	Amiodarone	Antiarrhythmic	**29**	Isradipine	Anti-hypertensive
**2**	Amitriptyline	Anti-depressant	**30**	Ketanserin	Anti-hypertensive
**3**	Astemizole	Antihistamine	**31**	Loratadine	Antihistaminic
**4**	Bepridil	Anti-anginal	**32**	Mesoridazine	Antipsychotic
**5**	Cetirizine	Antihistaminic	**33**	Mosapride	Peristaltic stimulant
**6**	Chlorpromazine	Antipsychotic	**34**	Nicotine	Anthelmintic
**7**	Ciprofloxacin	Anti-bacterial	**35**	Nifedipinde	Anti-anginal
**8**	Cisapride	Peristaltic stimulant	**36**	Nitrendipine	Anti-hypertensive
**9**	Clarithromycin	Anti-bacterial	**37**	Ondansetron	Antiemetic
**10**	Clozapine	Antipsychotic	**38**	Paroxetine	Anti-depressant
**11**	Cocaine	Topical anesthetic	**39**	Pentamidine	Anti-protozoal
**12**	Desipramine	Anti-depressant	**40**	Perhexiline	Diuretic
**13**	Diltiazem	Anti-anginal	**41**	Pimozide	Antipsychotic
**14**	Diphenhydramine	Antihistaminic	**42**	Procainamide	Cardiac depressant
**15**	Disopyramide	Cardiac depressant	**43**	Promethazine	Antihistaminic
**16**	Domperidone	Antiemetic	**44**	Prucalopride	Peristaltic stimulant
**17**	Doxepin	Anti-depressant	**45**	Pyrilamine	Antihistaminic
**18**	Droperidol	Antipsychotic	**46**	Quinidine	Cardiac depressant
**19**	Erythromycin	Anti-bacterial	**47**	Renzapride	Peristaltic stimulant
**20**	Fexofenadine	Antihistaminic	**48**	Sertraline	Anti-depressant
**21**	Flecainide	Cardiac depressant	**49**	Sotalol	Anti-anginal
**22**	Fluoxetine	Anti-depressant	**50**	Tamoxifen	Anti-estrogen
**23**	Glibenclamide	Anti-diabetic	**51**	Terodiline	Anti-anginal
**24**	Granisetron	Antiemetic	**52**	Terfenadine	Antihistaminic
**25**	Halofantrine	Anti-malarial	**53**	Thioridazine	Antipsychotic
**26**	Haloperidol	Antipsychotic	**54**	Verapamil	Anti-anginal
**27**	Hydroxyzine	Antihistaminic			
**28**	Imipramine	Anti-depressant			

**Table 2:** Computational analysis using ab initio HF/321G, Mulliken, NBO, and MKS of protonated drug.

	DRUG	Mulliken	NBO	MKS	MW	In Vitro IC_50_ (M)
**1**	Amiodarone	−0.79268	−0.54778	ND	644.36	0.1178 [49]; 1.0 [40] (ZF)
**2**	Amitriptyline	−0.80091	−0.23399	0.07330	278.42	10.0 [30]
**3**	Astemizole	−0.79546	−0.55224	−0.13528	459.59	0.001 [31]; 0.0009 [26]; 0.480 [28]
**4**	Bepridil	−0.78434	−0.55278	0.07330	367.56	0.55 [31]; 0.6 [40] (ZF)
**5**	Cetirizine	−0.78551	−0.54645	−0.21160	355.46	>30 [28]
**6**	Chlorpromazine	−0.79922	−0.55743	0.06496	319.88	1.47 [30]
**7**	Ciprofloxacin	−0.80141	−0.70264	−0.37912	331.35	966 [31]
**8**	Cisapride	−0.79574	−0.54571	−0.04759	466.96	0.0067 [30]; 0065 [29]; 0.0024 [27]; 0.018 [52], 0.0094 [38]
**9**	Clarithromycin	−0.79433	−0.16888	−0.03182	748.98	720 [31]
**10**	Clozapine	−0.79097	−0.55892	0.14914	293.39	0.32 [30]
**11**	Cocaine	−0.74627	−0.55506	−0.14587	304.37	7.2 [30]
**12**	Desipramine	−0.81479	−0.70178	−0.08777	267.40	1.39 [30]
**13**	Diltiazem	−0.80148	−0.55903	0.01192	415.54	17 [31]; 17.3 [30]
**14**	Diphenhydramine	−0.80027	−0.56729	0.08497	256.37	2.7 [45]
**15**	Disopyramide	−0.79334	−0.54725	−0.33518	312.44	25.7 [42]
**16**	Domperidone	−0.82296	−0.56875	0.10563	426.93	0.16 [31]
**17**	Doxepin	−0.80126	−0.12408	−0.05770	280.39	6.5 ± 1.4 [37]
**18**	Droperidol	−0.80410	−0.54607	0.10986	394.47	0.032 [31]
**19**	Erythromycin	−0.81967	−0.17136	0.00521	734.95	>>10 [31]
**20**	Fexofenadine	−0.82284	−0.57374	−0.17395	501.67	214 [51]
**21**	Flecainide	−0.81180	−0.70384	−0.37655	415.36	1.49 [48]
**22**	Fluoxetine	−0.81306	−0.70083	−0.25308	310.34	3.1 [31]
**23**	Glibenclamide	−0.87870	−0.75840	−0.79220	495.02	74 [29]
**24**	Granisetron	−0.79173	−0.54915	−0.28540	313.43	3.73 [30]
**25**	Halofantrine	−0.80869	−0.54401	0.72309	501.44	0.2 [31]; 0.196 [27]
**26**	Haloperidol	−0.80358	−0.54984	−0.04346	376.88	0.0268 [27]; 0.0281 [29]; 0.063 [36]
**27**	Hydroxyzine	−0.79906	−0.55469	−0.02649	375.92	0.18 ± 0.02 [42]
**28**	Imipramine	−0.79841	−0.55672	0.03611	281.42	3.4 [30]
**29**	Isradipine	−0.94620	−0.55204	−0.14381	386.43	>10 [31]
**30**	Ketanserin	−0.79680	−0.55016	−0.22219	396.44	0.38 ± 0.04 [52]
**31**	Loratadine	−0.87400	−0.62765	−0.37319	383.90	0.17 [31]; 0.173 [30]; 4.0 [35]
**32**	Mesoridazine	−0.78787	−0.54659	−0.06959	403.59	0.32 [30]
**33**	Mosapride	−0.78990	−0.55123	0.12172	422.91	4.8 [38]
**34**	Nicotine	−0.78335	−0.55905	−0.01477	163.24	244.8 [30]
**35**	Nifedipine	−1.18249	−0.62345	0.01959	347.35	>>50 [31]
**36**	Nitrendipine	−0.97682	−0.70166	−0.12265	361.38	>>10 [31]
**37**	Ondansetron	−0.87858	−0.60831	0.59556	280.35	0.81 [31]; 0.81 [30]
**38**	Paroxetine	−0.82135	−0.70448	−0.39771	330.38	0.45 [46]
**39**	Pentamidine	−0.90198	−0.85219	−0.93083	341.44	5–8 [34]
**40**	Perhexiline	−0.81614	−0.69626	−0.46765	278.50	7.8 [30]
**41**	Pimozide	−0.82589	−0.57080	−0.00933	462.57	0.018 [31]; 0.0546 [30]; 0.018 [29]; 0.015 [40]
**42**	Procainamide	−0.79334	−0.55222	−0.38558	236.34	139 [33]; 310 [40]
**43**	Promethazine	−0.79435	−0.55243	−0.05435	285.44	0.73 [41]
**44**	Prucalopride	−0.78997	−0.54834	−0.06131	368.89	0.57 [27]; 4.1 [42]
**45**	Pyrilamine	−0.80175	−0.56007	−0.18997	286.40	6.0 [35]
**46**	Quinidine	−0.80419	−0.55906	−0.08513	325.43	0.32 [28]
**47**	Renzapride	−0.78612	−0.55461	0.03873	324.83	0.018 [27]
**48**	Sertraline	−0.80158	−0.69988	−0.34017	307.24	0.210 [35]; 0.70 [46]
**49**	Sotalol	−0.80926	−0.69660	−0.52732	273.38	343 [52]
**50**	Tamoxifen	−0.79866	−0.56765	0.17060	372.53	1.2 [47]
**51**	Terodiline	−0.79403	−0.67800	−0.71526	282.45	0.375 +/− 0.004 [36]
**52**	Terfenadine	−0.79399	−0.54866	−0.00184	472.69	0.213 [30]; 0.300 [28]; ≤ 0.052 [35]; 0.165 and 0.031 [51]
**53**	Thioridazine	−0.81089	−0.57002	0.20288	371.59	0.0332 [30]; 0.0357 [29]; 0.390 [35]; 0.033 [40] (ZF)
**54**	Verapamil	−0.80749	−0.55449	−0.05016	455.62	0.8 [30]; 0.143 [29]; 0.214 [51]; 0.14 [39]
	ZF = Zebrafish model					

**Table 3:** Second set of compounds that may prolong QT and/or induce Torsades de Pointes.

	Drug	Classification	Drug		Classification
**55**	Azimilide	Class ΙΙΙ antiarrhythmic	**79**	MDL 74,156	5-HT3 antagonist
**56**	Bilastine	2nd Gen antihistamine	**80**	Nicardipine	Anti-Anginal
**57**	Clozapine-*N*-oxide	Metabolite	**81**	Norastemizole	Antihistamine
**58**	Desmethyl-clozapine	Metabolite	**82**	Prenylamine	Calcium channel blocker
**59**	O-desmethyl-astemizole	Metabolite	**83**	Propafenone	Antiarrhythmic
**60**	Ebastine	2nd Gen H1 antagonist	**84**	Rupatadine	2nd Gen antihistamine
**61**	Carebastine	Active metabolite	**85**	Risperidone	Antipsychotic
**62**	Olanzapine	Antipsychotic	**86**	OHRisperidone	Metabolite
**63**	Desmethyl-olanzapine	Antipsychotic Metabolite	**87**	Sertindole	Antipsychotic
**64**	2-OH-methyl-olanzapine	Antipsychotic Metabolite	**88**	Sildenafil	Erectile dysfunction
**65**	2-Carboxy-Me-olanzapine	Antipsychotic Metabolite		Sparfloxacin	Antibiotic
**66**	CJ-033466	5-HT4 partial agonist	**90**	Sumatriptan	Anti-migraine
**67**	Dofetilide	Treat atrial flutter	**91**	Ziprasidone	Antipsychotic
**68**	Dolasetron	5-HT3 antagonist			
**69**	DSP-6952	5-HT_4_ Receptor Agonist			
**70**	Chlorphenamine	Antihistamine			
**71**	E-4031	Class III Antiarrhythmic			
**72**	Endoxifen	Metabolite			
**73**	Gatifloxacin	Antibiotic			
**74**	Lidoflazine	Calcium channel blocker			
**75**	Levofloxacin	Antibiotic			
**76**	Mibefradil	for hypertension			
**77**	Mexiletine	Antiarrhythmic agent			
**78**	MK-499	Class III Antiarrhythmic			

**Table 4:** Computational analysis using ab initio HF/321G, Mulliken, NBO, molecular weight, and cLogP of protonated drug compound set two.

Comp. #	Drug	Mulliken	NBO	MW	cLogP	Estimate	IC50
**55-A**	azimilide N1	−0.77112	−0.5513	457.95	2.35	0.1–1.0	0.58; 0.6 [1]
**55-B**	azimilide N2	−0.76740	−0.5577	---	---	---	---
**56**	Bilastine	−0.82271	−0.5939	463.61	1.95	>4.0	6.5–17.1 [51]
**57**	Clozapine-N-oxide	−0.53326	−0.2343	342.82	3.71	>7.0	133.3 [30]
**58**	Desmethyl-clozapine	−0.78796	−0.7106	312.80	3.46	2.0–4.0	4.49 [30]
**59**	O-DesMe-astemizole	−0.77213	−0.5507	444.54	5.50	<0.1	0.01 [54]
**60**	Ebastine	−0.76819	−0.5477	469.66	6.94	<0.1	0.014; 0.30 [49]
**61**	Carebastine	−0.74827	−0.6089	499.64	2.83	>4.0	No data
**62**	Olanzapine	−0.76581	−0.5574	312.43	3.01	0.1–1.0	0.231 [30]
**63**	Desmethyl-olanzapine	−0.78776	−0.7104	298.41	2.75	>4.0	14.2 [30]
**64**	2-OH-Me-olanzapine	−0.76572	−0.5574	328.43	1.47	0.1–1.0	0.23, 11.6 [39]
**65**	2-carboxy-olanzapine	−0.76382	−0.5566	342.42	0.27	>4.0	No data
**66**	CJ−033466	−0.77129	−0.5458	377.91	3.26	1.0–2.0	2.6 [38]
**67**	Dofetilide	−0.78606	−0.5572	441.56	1.99	<0.1	0.007 [52]
**68**	Dolasetron	−0.77293	−0.5571	324.37	1.18	4.0–6.0	5.95 [29]
**69**	DSP-6952	−0.76758	−0.5489	454.95	−0.26	0.1–1.0	0.271 [50]
**70**	Chlorphenamine	−0.77488	−0.5540	274.12	3.15	1.0–2.0	13 [35]
**71**	E-4031	−0.77456	−0.5493	401.52	1.90	<0.1	0.077 [29]
**72**	Endoxifen	−0.82062	−0.7235	373.49	5.56	2.0–4.0	1.6 [47]
**73**	Gatifloxacin	−0.83650	−0.7429	375.39	−0.27	>6.0	50 [55]
**74-A**	Lidoflazine N1	−0.76575	−0.5478	491.62	4.79	<0.1	≤0.037 [35]
**74-B**	Lidoflazine N2	−0.79747	−0.5730	---	---	---	---
**75**	Levofloxacin	−0.82808	−0.6079	361.37	−0.51	>4.0	915 [55]
**76**	Mibefradil	−0.77723	−0.5471	495.63	6.36	1.0–2.0	1.43 [29]
**77**	Mexiletine	−0.84670	−0.7498	179.26	2.57	>6.0	>> 10 [31]
**78**	MK-499	−0.77257	−0.5430	467.58	1.63	<0.1	0.032 [31]; 0.090–0.107
**79**	MDL 74,156	ND	ND	326.39	0.74	4.0–6.0	12 [31]
**80**	Nicardipine	−0.97746	−0.55123	480.54	5.23	>10	No data
**81**	Norastemizole	−0.78844	−0.7079	324.18	3.63	<0.1	0.028 [54]
**82**	Prenylamine	−0.79110	−0.6951	329.48	5.80	1.0–2.0	0.59 [35]
**83**	Propafenone	−0.82310	−0.7145	341.44	3.64	1.0–2.0	2.0 [35]
**84**	Rupatadine	−0.75723	−0.5493	415.96	5.06	>4.0	8.1 [51]
**85**	Risperidone	−0.77038	−0.5479	410.48	2.71	<1.0	0.14 [31], 0.148 [30]
**86**	9-hydroxy risperidone	−0.77050	−0.5480	426.48	1.07	<1.0	1.3 [29]
**87**	Sertindole	−0.82424	−0.5942	440.94	5.27	<0.1	0.024 [31]
**88**	Sildenafil	−0.76843	−0.5594	474.58	1.98	4.0–6.0	3.3 [30]
**89**	Sparfloxacin	−0.80527	−0.7131	392.4	−0.60	>4.0	18 [31]
**90**	Sumatriptan	−0.81664	−0.57292	396.42	0.74	Est	No data
**91**	Ziprasidone	−0.76987	−0.5508	412.94	4.21	0.1–1.0	0.15 [31]

## Data Availability

Data is summarized in Supplementary Materials Section.

## Supplementary Materials

The following supporting information can be downloaded at: https://www.mdpi.com/article/10.3390/ijms231810732/s1.
