# Supplementary material for: New Insights into Ion Channels: Predicting hERG-Drug Interactions
Source: Int J Mol Sci. 2022 Sep 14;23(18):10732. doi: 10.3390/ijms231810732 (PMC9503154; doi:10.3390/ijms231810732)
Supplement: Supplementary file 1 [file ijms-23-10732-s001.zip › ijms-1867594-supplementary.pdf]

Article

Supplementary Materials Section

New insights into ion channels: Predicting hERG-Drug Interactions

Michael Fitzpatrick Wempe [1,2]

[1] Department of Pharmaceutical Sciences, Skaggs School of Pharmacy and Pharmaceutical Sciences, University of Colorado—Anschutz Medical Campus, Aurora, CO 80045, USA; michael.wempe@cuanschutz.edu; Tel.: +1-303-724-8982
[2] University of Colorado Cancer Center, University of Colorado—Anschutz Medical Campus, Aurora, CO 80045, USA

Abstract: Drug-induced long QT syndrome can be a very dangerous side effect of existing and developmental drugs. In this work, a model proposed two decades ago addressing the ion specificity of potassium channels is extended to the human ether-à-gogo gene (hERG). hERG encodes the protein that assembles into the potassium channel responsible for the delayed rectifier current in ventricular cardiac myocytes that is often targeted by drugs associated with QT prolongation. The predictive value of this model can guide a rational drug design decision early in the drug development process and enhance NCE (New Chemical Entity) retention. Small molecule drugs containing a nitrogen that can be protonated to afford a formal +1 charge can interact with hERG to prevent the repolarization of outward rectifier currents. Low-level ab initio calculations are employed to generate electronic features of the drug molecules that are known to interact with hERG. These calculations were employed to generate structure–activity relationships (SAR) that predict whether a small molecule drug containing a protonated nitrogen has the potential to interact with and inhibit the activity of the hERG potassium channels of the heart. The model of the mechanism underlying the ion specificity of potassium channels offers predictive value toward optimizing drug design and, therefore, minimizes the effort and expense invested in compounds with the potential for life-threatening inhibitory activity of the hERG potassium channel.

Keywords: potassium channel; human ether-à-gogo-related gene (hERG); structure–activity relationships (SAR); ab initio calculations; drug development; long QT syndrome; *Torsades de Pointes*

Table S1 G98W HF/321G and Chem3D property server data

Ion		MW (amu)	---- CONNOLLY ---- AA (°A²)	MA (°A²)	SEV (°A³)	Electron Density	Radius (°A)
Lithium	Li^+	6.94	70.64	11.85	3.84	1.97	0.60
Magnesium	Mg^{+2}	24.31	81.97	16.74	6.44	10.04	0.65
Calcium	Ca^{+2}	40.08	89.85	20.40	8.66	19.87	0.99
Sodium	Na^+	22.99	90.73	20.82	8.93	10.15	0.95
Water	H_2O	18.02	96.91	23.48	10.40	--------	-----
Potassium	K^+	39.10	112.50	31.85	16.90	19.64	1.33
Rubidium	Rb^+	85.47	118.75	35.21	19.65	43.82	1.48
	$H_2O{\cdot}Na^+$	41.01	131.77	39.27	19.59	10.22	-----
	$3H_2O{\cdot}Li^+$	60.99	195.25	69.76	37.89	2.12	-----

AA = Accessible Area; MA = Molecular Area; SEV = Solvent-Excluded Volume

Atomic Charge from G98W Output, other data obtained from the Chem3D Property server

Table S2 Protonated Amines, pKa, MW, atomic charges on amine and CLogP

Amine	pK_a	MW	CMA	Mulliken AM1	Mulliken HF/321G	NBO HF/321G	MKS HF/321G	ClogP
$Bu_2NH_2^+$	11.25	130.25	189.49	-0.2332	-0.8184	-0.6949	-0.1290	3.306
Piperidine-NH_2^+	11.12	86.16	113.95	-0.2339	-0.8038	-0.7056	-0.4082	1.389
$Et_2NH_2^+$	10.94	74.15	115.36	-0.2374	-0.8034	-0.7029	-0.5221	1.190
$EtNH_3^+$	10.75	46.09	73.33	-0.2932	-0.8339	-0.8620	-0.6202	-0.095
Et_3NH^+	10.75	102.20	147.36	-0.1805	-0.7875	-0.5555	-0.3476	1.605
$Me_2NH_2^+$	10.73	46.09	76.66	-0.2331	-0.8160	-0.7126	-0.1385	0.132
$MeNH_3^+$	10.64	32.07	52.68	-0.2888	-0.8428	-0.8673	-0.3688	-0.624
Bu_3NH^+	9.93	186.36	258.90	-0.1743	-0.8101	-0.5454	0.6550	4.779
Me_3NH^+	9.79	60.12	96.86	-0.1788	-0.7986	-0.5666	0.0430	0.018
Benzyl-NH_3^+	9.35	108.16	136.37	-0.2929	-0.8220	-0.8576	-0.4203	1.144
NH_4^+	9.26	18.04	25.79	-0.3460	-0.8805	-1.0300	-0.8721	0.330
Phenyl-NEt_2H^+	6.65	150.25	183.35	-0.1236	-0.8739	-0.5438	-0.4171	3.365
Phenyl-NMe_2H^+	5.80	122.19	149.44	-0.1207	-0.8819	-0.5475	-0.3234	2.307
Pyridinium ion	5.29	80.11	99.13	-0.2537	-0.8928	-0.5738	-0.2407	0.645
Phenyl-NH_3^+	4.63	94.14	107.68	-0.2281	-0.9282	-0.8447	-0.9501	0.811
$Phenyl_2$-NH_2^+	1.00	170.25	184.02	-0.1887	-0.9798	-0.6744	-0.9532	3.186
$Phenyl_3$-NH^+	-5.00	246.33	251.17	-0.0148	-1.0495	-0.5158	-1.0794	5.735

Figure S1 hERG model test compounds

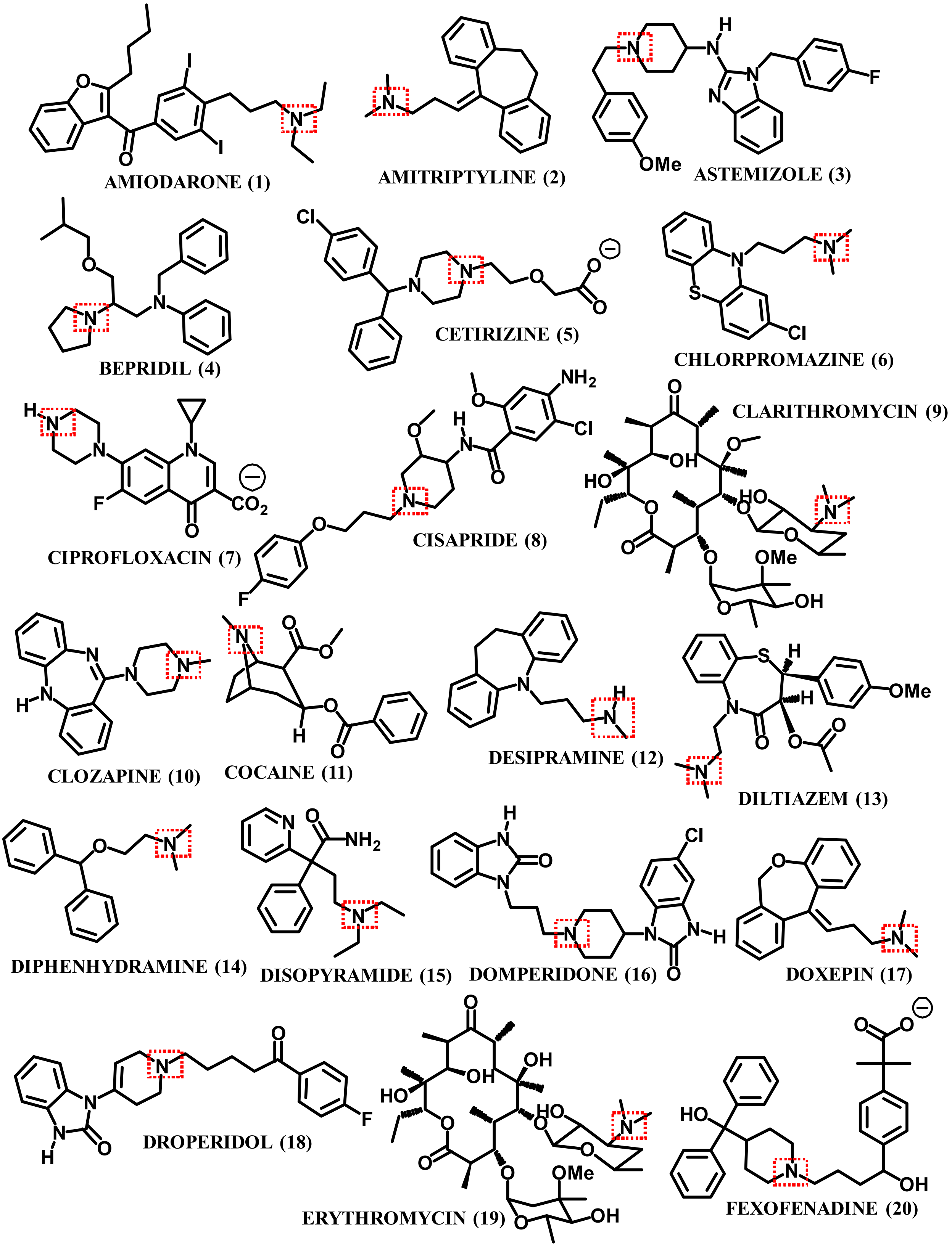

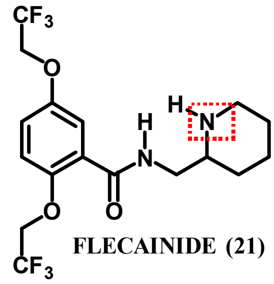

FLECAINIDE (21)

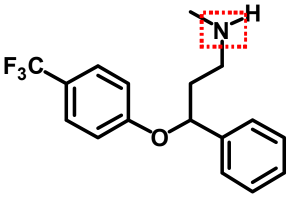

FLUOXETINE (22)

GLIBENCLAMIDE (23)

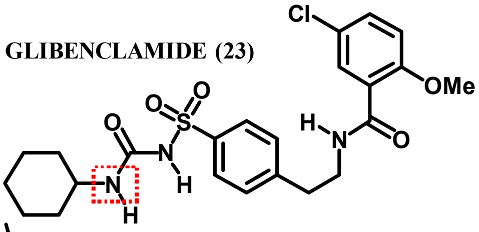

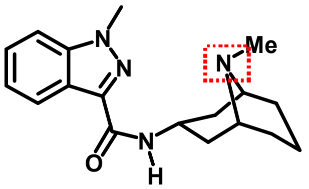

GRANISETRON (24)

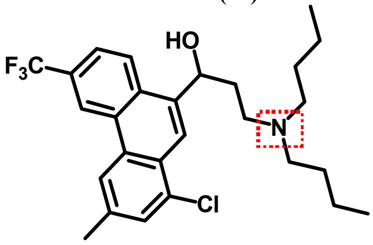

HALOFANTRINE (25)

HALOPERIDOL (26)

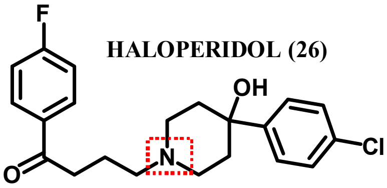

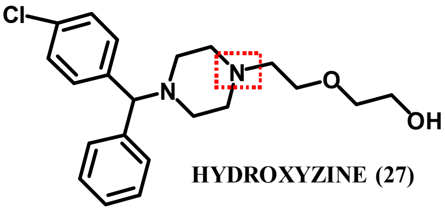

HYDROXYZINE (27)

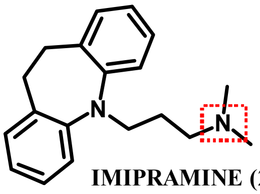

IMIPRAMINE (28)

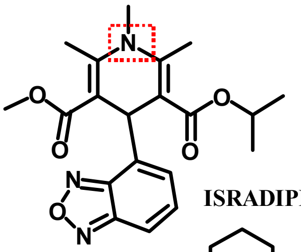

ISRADIPINE (29)

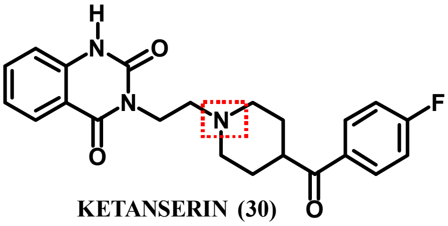

KETANSERIN (30)

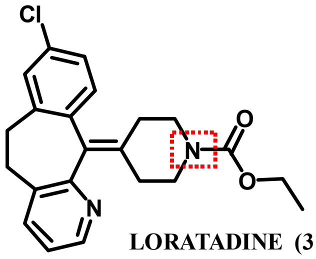

LORATADINE (31)

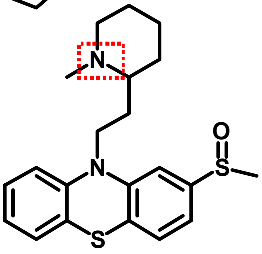

MESORIDAZINE (32)

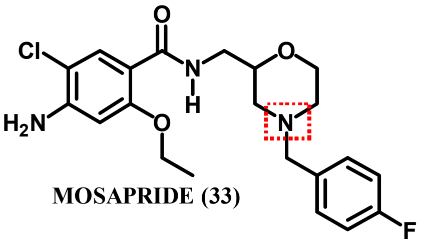

MOSAPRIDE (33)

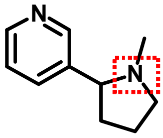

NICOTINE (34)

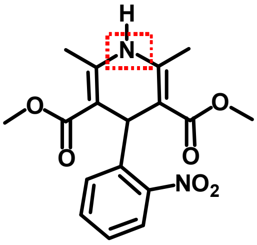

NIFEDIPINE (35)

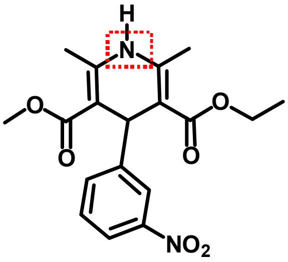

NITRENDIPINE (36)

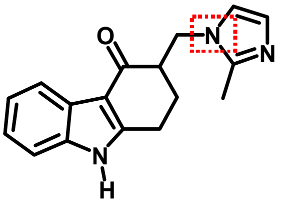

ONDANSETRON (37)

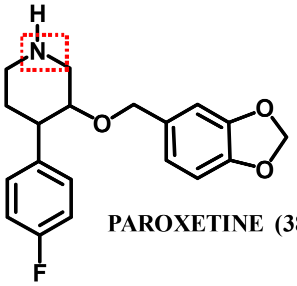

PAROXETINE (38)

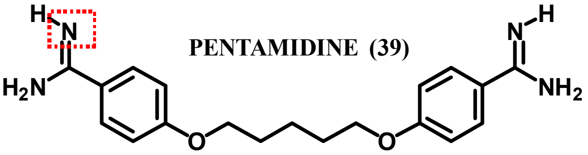

PENTAMIDINE (39)

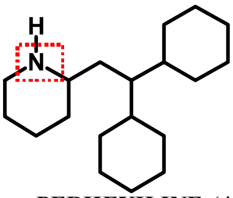

PERHEXILINE (40)

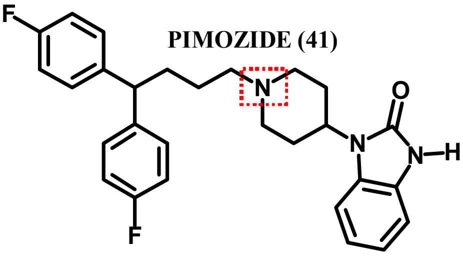

PIMOZIDE (41)

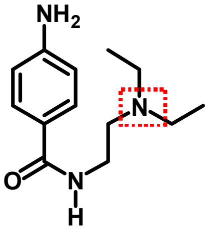

PROCAINAMIDE (42)

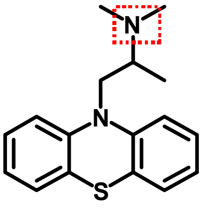

PROMETHAZINE (43)

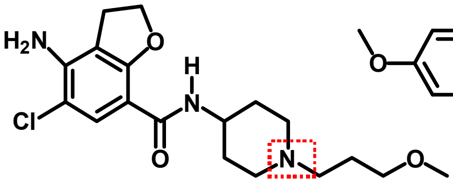

PRUCALOPRIDE (44

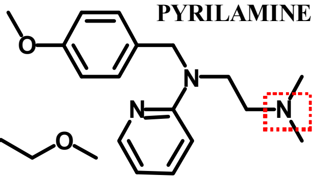

PYRILAMINE (45)

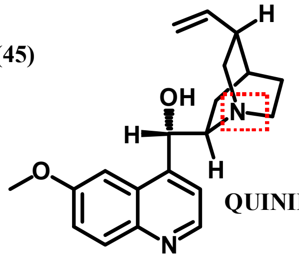

QUINIDINE (46)

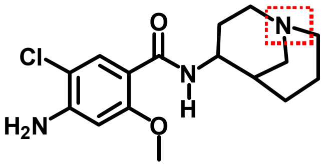

RENZAPRIDE (47)

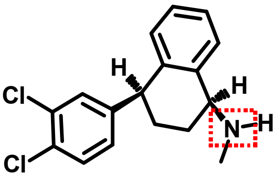

SERTRALINE (48)

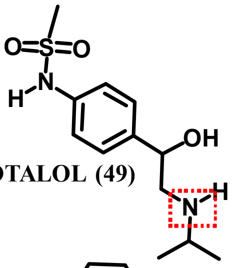

SOTALOL (49)

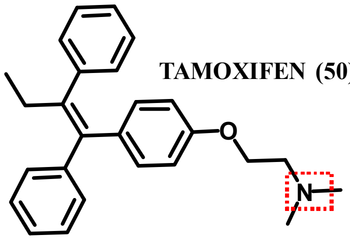

TAMOXIFEN (50)

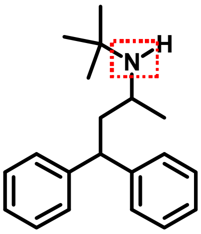

TERODILINE (51)

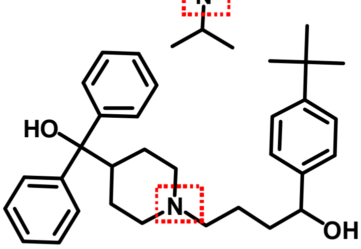

TERFENADINE (52)

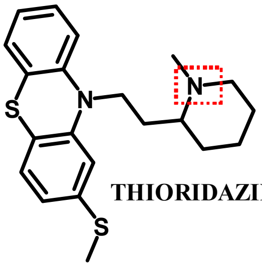

THIORIDAZINE (53)

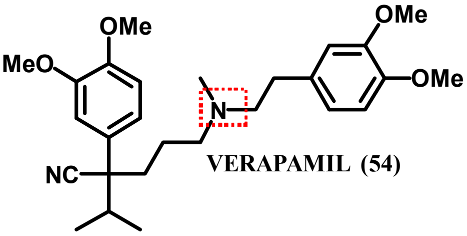

VERAPAMIL (54)

Figure S2 Compounds from **Table 1** HF/321G NBO after Q1-Q4; IC_{50} vs NBO

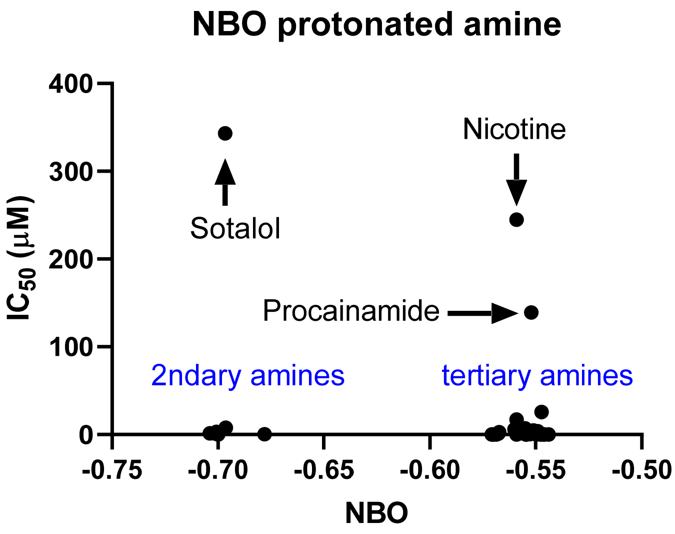

Figure S3 Compounds from **Table 1** HF/321G NBO after nicotine, sotalol, and procainamide removed; IC_{50} vs NBO

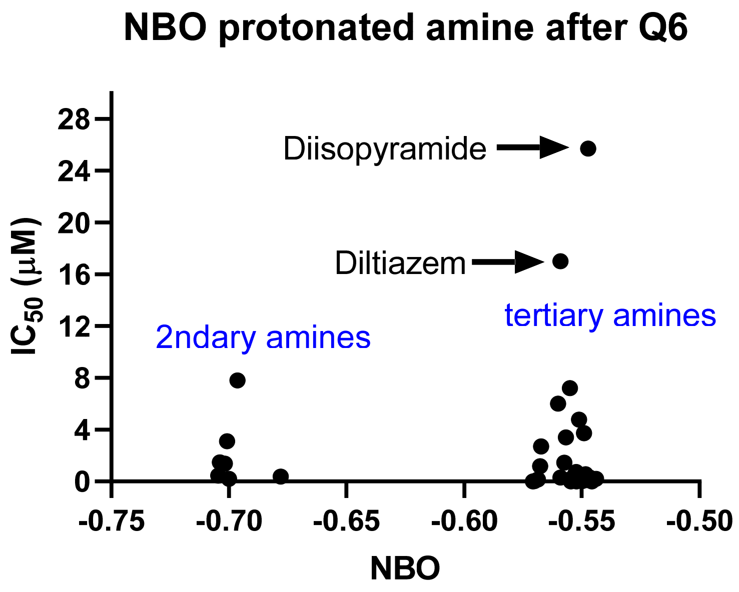

Table S3 Summary of Q6 from learning set

IC50 μM	2 Aromatic Rings	*N* Alkyl<br>2ndary amines;	distance from amine	Direction
0.21	Sertraline	Secondary; methyl-cyclohexyl (109 amu)	2 and 4 BDS, Sep by 1 bond	Same
0.375	Terodiline	Secondary; tert-butyl-isobutyl (128 amu)	3 and 3 BDS, Sep by 1 bond	Same
0.45	Paroxetine	Secondary; Piperidine-3-O-ethyl (100 amu)	3 and 4 BDS, Sep by 4 BDS	Same
1.39	Desipramine	Secondary; Methyl-propyl (87 amu)	4 and 4 BDS, Sep by 1 bond	Same
3.1	Fluoxetine	Secondary; Methyl-4-O-butyl (88 amu)	3 and 4 BDS, Sep by 2 BDS	Same

IC50 μM	2 Aromatic Rings	Tertiary Amines	distance from amine	Direction
0.0067	Cisapride	Tertiary; 4-O-butyl, pip-3-methoxy (172 amu)	4 and 5 BDS, Sep by 10 BDS	Opposite
0.0268	Haloperidol	Tertiary; dibutyl-3-OH-propyl (142 amu)	3 and 4 BDS, Sep by 8 BDS	Opposite
0.033	Thioridazine	Tertiary;methyl-2-ethyl-pip (141 amu)	4 and 4, Sep 1 BDS	Same
0.18	Hydroxyzine	Tertiary; -CH-pip-Et-O-EtOH (141 amu)	4 and 4 BDS, Sep by 1 bond	Same
0.21	Verapamil	Tertiary; Me-Et-4-CN-5-me-hexane (111 amu)	2 and 4 BDS, Sep by 7 BDS	Same
0.32	Quinidine	Tertiary; bicyclo2:2:2 -CH-2-allyl (155 amu)	2 and 3 BDS, Sep by 0 BDS	Same
0.55	Bepridil	Tertiary; 3-O-5-Me-hexyl;3-*N*-butyl (128 amu)	3 and 4 BDS, Sep by 2 BDS	Opposite
2.7	Diphenylhydramine	Tertiary; diMe-3-O-butyl (102 amu)	4 & 4 BDS, Sep by1 BDS	Same
3.4	Imipramine	Tertiary; dimethyl-propyl (102 amu)	4 and 4 BDS, Sep by 1 bond	Opposite
3.7	Granisetron	Tertiary; Methyl-bicyclo-3:3- (127 amu)	5 and 6 BDS, Sep by 0 BDS	Same
4.8	Mosapride	Tertiary; -CH_2-morp-3-CH_2NHCO- (114 amu)	1 and 5 BDS, Sep by 7 BDS	Opposite
6.0	Pyrilamine	Tertiary; Dimethyl-3-N-butyl (86 amu)	3 and 4 BDS, Sep by 2 BDS	Same
17	Diltiazem	Tertiary; Dimethyl-ethyl (87 amu)	3 and 6 BDS, Sep by 2 BDS	Same
25.7	Di-isopyramide	Tertiary; DiEt-propyl (111 amu)	3 & 3 BDS, Sep by 1 BDS	Same

IC50 μM	3 aromatic rings	N Alkyl	atom distance from amine	Direction

0.032	Droperidol	Tert; 5-oxopent-2-Et-cyHex (138 amu)	3,4 and 5 BDS, Sep 9 BDS	Opposite
0.117	Amiodarone	Tertiary; diethylpropyl (115 amu)	3, 8 and 9 BDS, Sep 1 and 0 BDS	Same
0.20	Halofantrine	Tertiary; dibut-3-OH-prop (187 amu)	3,4, and 5 BDS, Sep 0 BDS	Same
0.213	Terfenadine	Tert; 4-OH-but,4CHOH)pip (185 amu)	4,4 and 4 BDS, Sep 9 and 9 BDS	Opposite
0.32	Clozapine	Tertiary; Me-4-R-piperazine (100 amu)	3,4, and 5 BDS, Sep 0 BDS	Same
0.32	Mesoridazine	Tertiary; Me-2-Et-piperidine (127 amu)	3,4, and 4 BDS, Sep 0 BDS	Same
0.38	Ketanserin	Tertiary; 1-Et-4-R-piperidine (112 amu)	2,3, and 4 BDS, Sep 7 BDS	Opposite
0.73	Promethazine	Tertiary; dimethyl-2-propyl (87 amu)	2,3 and 3 BDS, Sep 0 BDS	Same
1.20	Tamoxifen	Tertiary; diMe-3-O-propyl (89 amu)	3, 8 and 9 BDS, Sep 1 and 2 BDS	Same
1.47	Chlorpromazine	Tertiary; dimethylpropyl (87 amu)	3, 4 and 4 BDS, Sep 0 BDS	Same

IC50 μM	Four aromatic rings	N Alkyl	atom distance from amine	Direction
0.001	Astemizole	Tertiary; Et-4R-piperidine (112 amu)	2, 4, 6 and 7, Sep by 7 and 10 BDS	Opposite
0.018	Pimozide	Tertiary; Bu-4R-piperazine (139 amu)	4,4 and 3,4 Sep by 8 BDS	Opposite
0.16	Domperidone	Tert; Propyl-4R-piperazine (126 amu)	3,4 and 3,4 Sep by 7 BDS	Opposite

Figure S4 Terfenadine versus fexofenadine

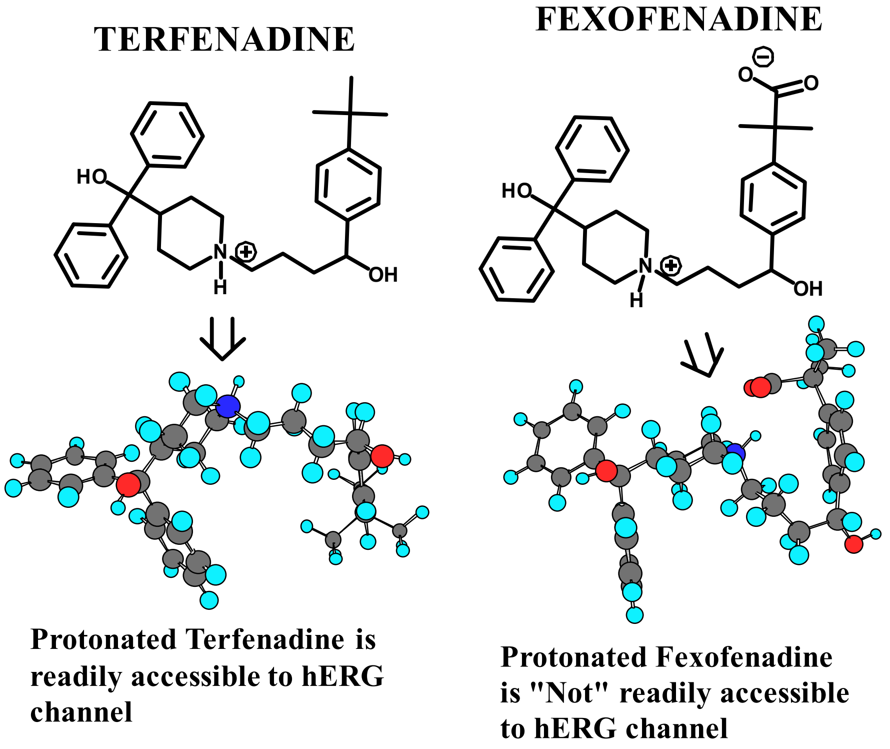

Figure S5 Cisapride, mosapride, prucalopride and renzapride

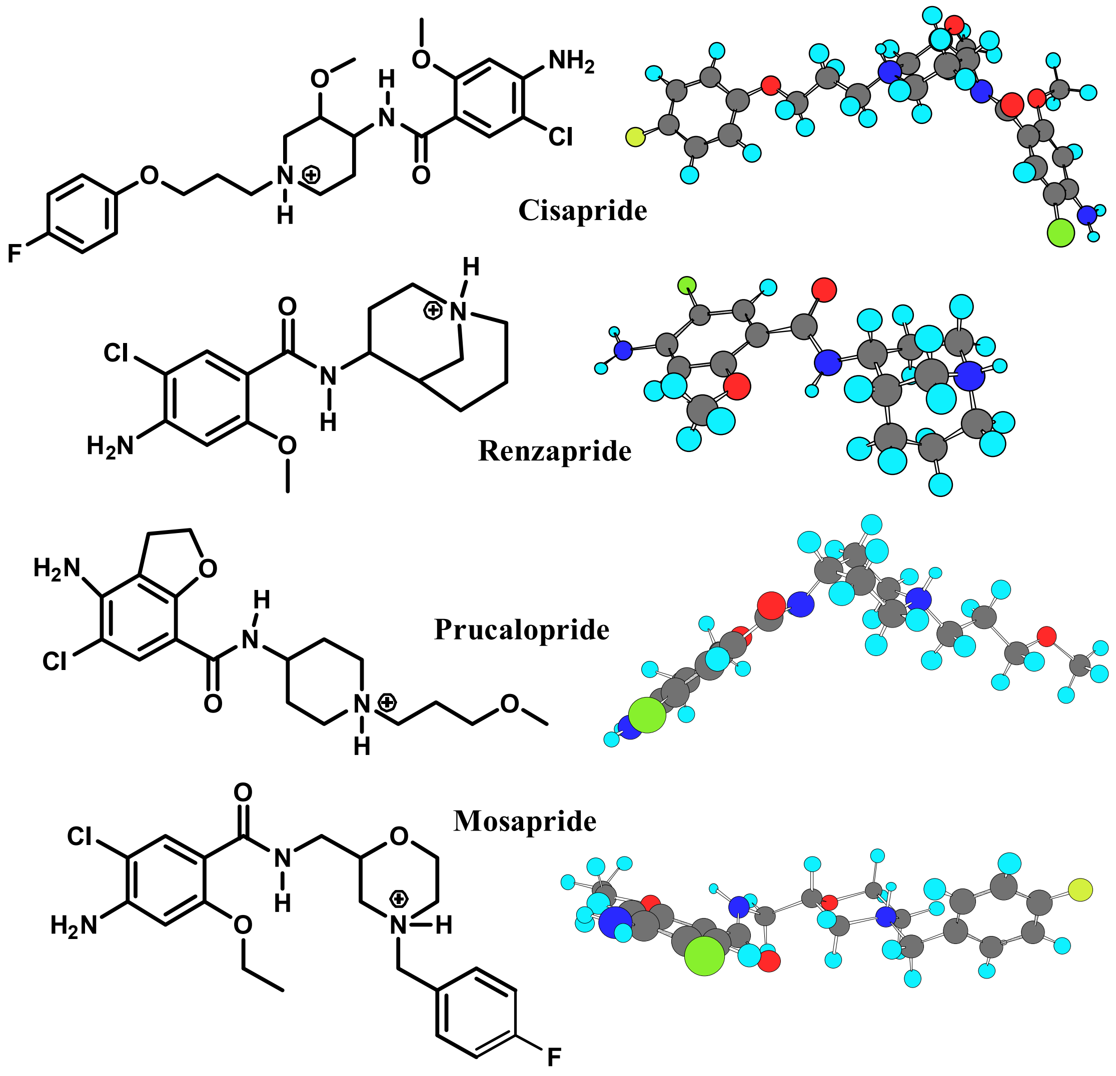

Figure S6 Additional hERG compounds; Set Two

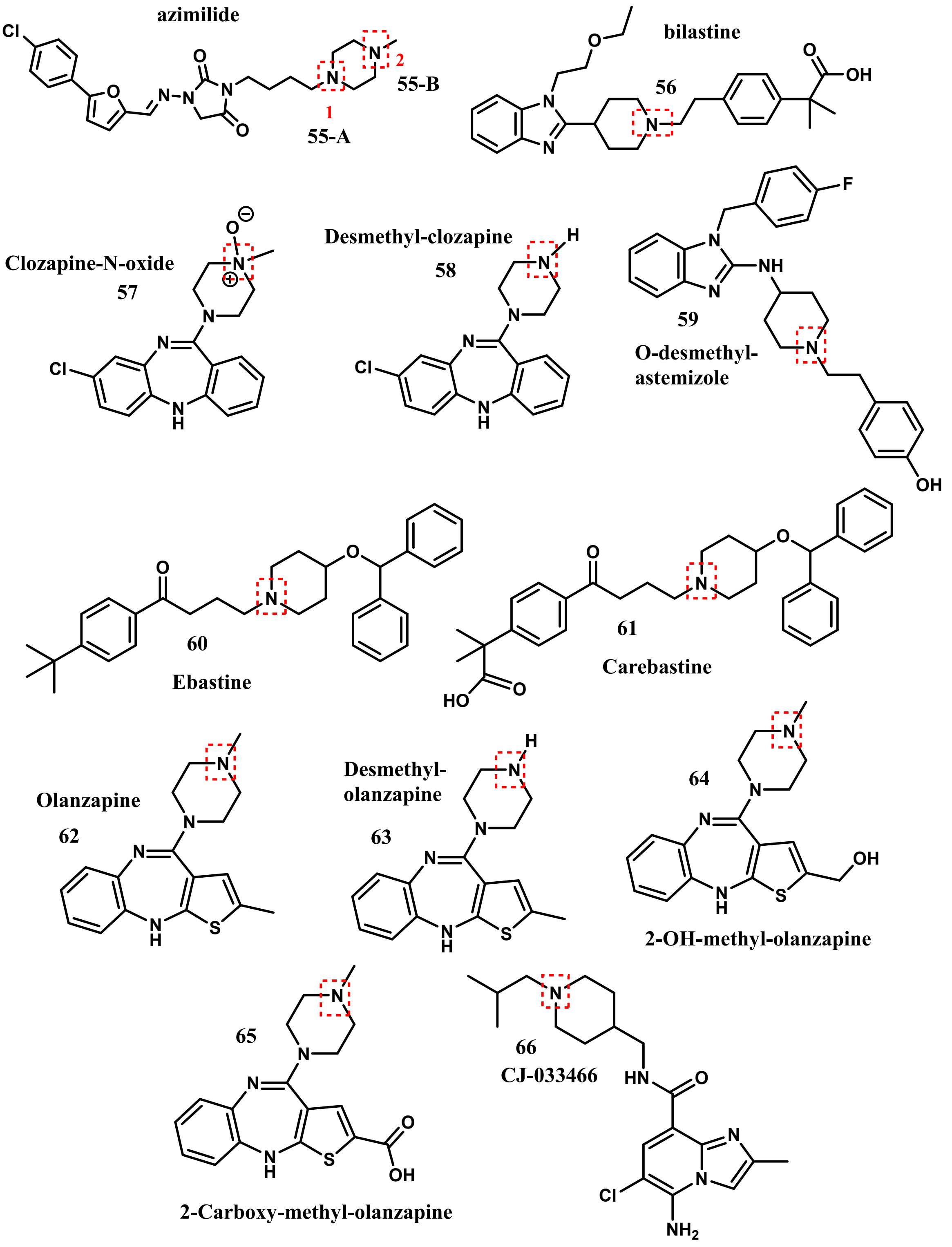

67 dofetilide

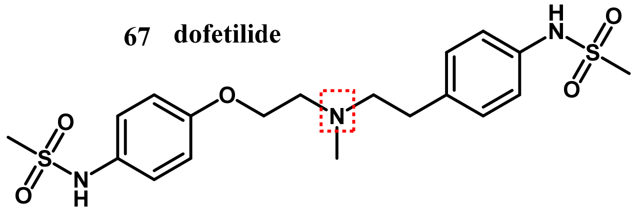

68 Dolasetron

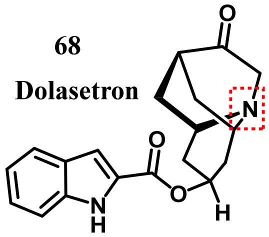

69 DSP-6952

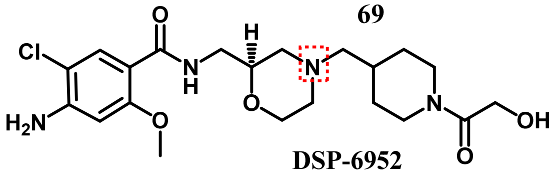

Chlorphenamine 70

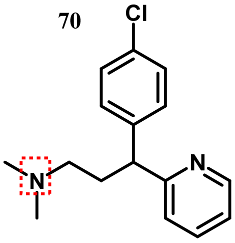

71 E-4031

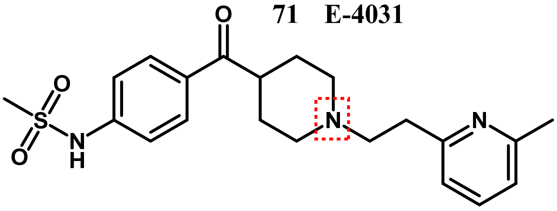

72 Endoxifen

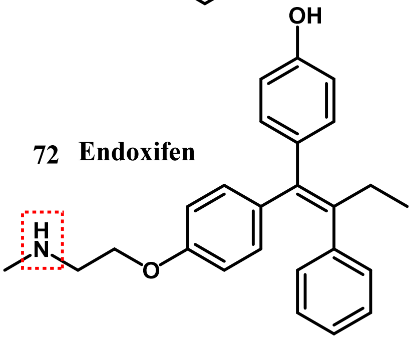

73 Gatifloxacin

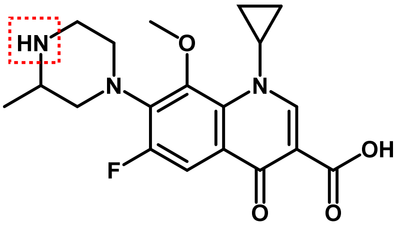

74 Lidoflazine

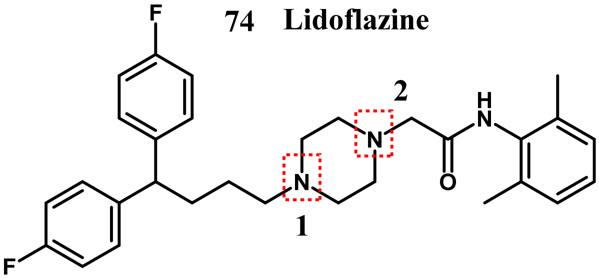

75 levofloxacin

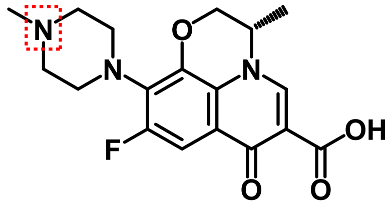

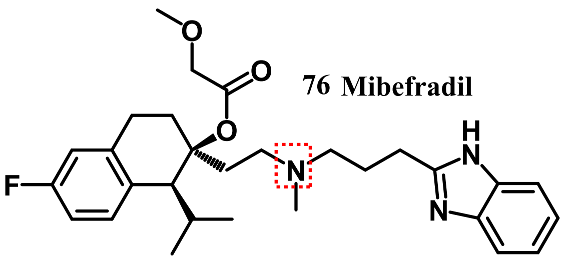

76 Mibefradil

77 Mexiletine

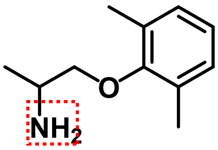

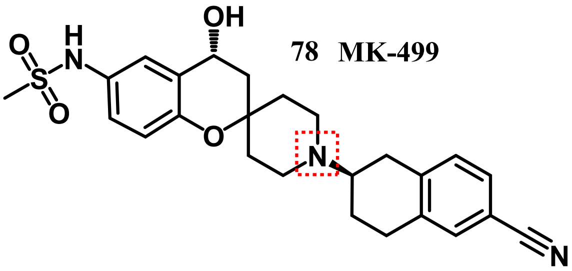

78 MK-499

79

MDL 74,156

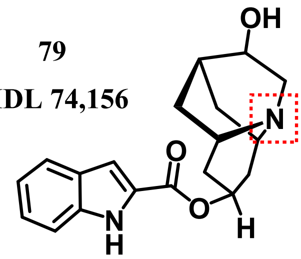

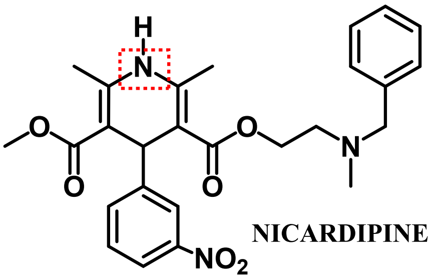

NICARDIPINE (80)

norastemizole

81

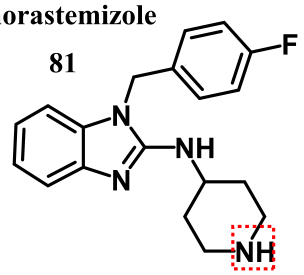

Prenylamine

82

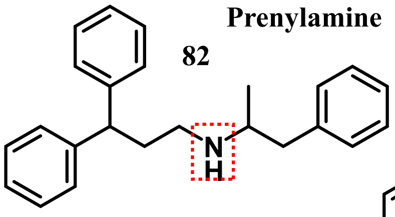

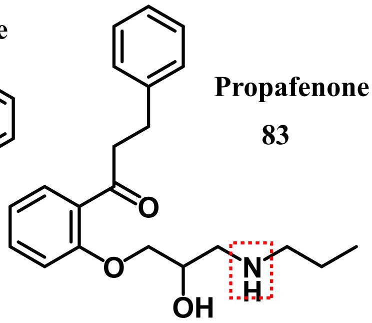

Propafenone

83

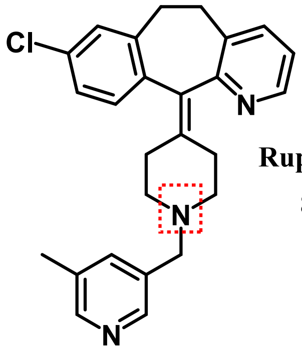

Rupatadine

84

Risperidone

85

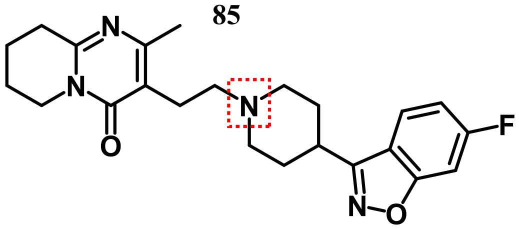

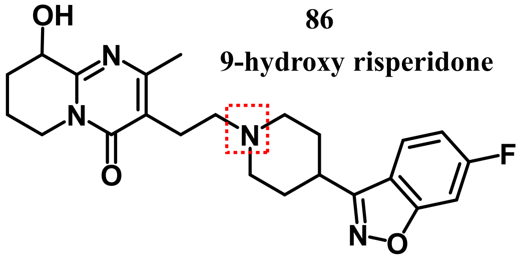

86

9-hydroxy risperidone

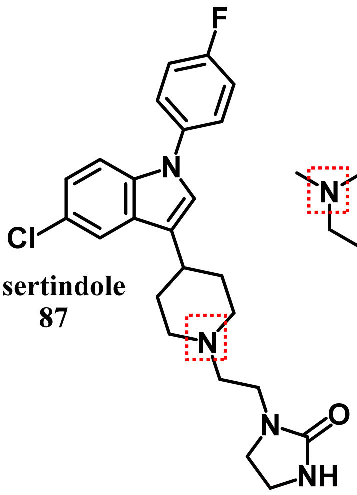

sertindole
87

sildenafil
88

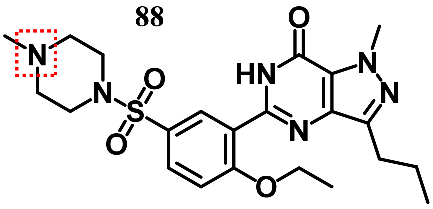

89 sparfloxacin

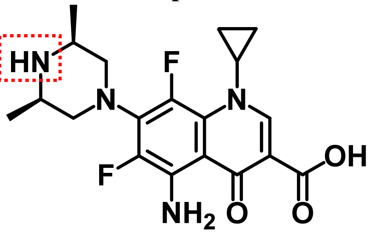

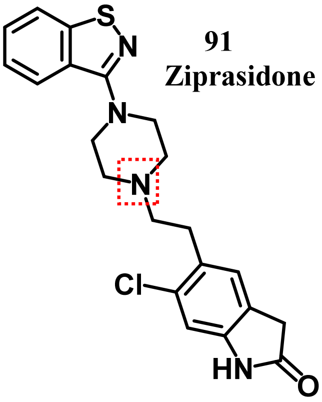

91
Ziprasidone

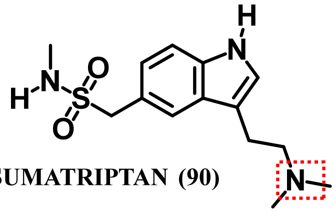

SUMATRIPTAN (90)

Scheme S1 hERG Channel prediction flow-chart for compound set two

hERG Channel Flow-chart

Q1: From the test set, which compounds do not contain an amine?

N/A

Q2: From the amines, is the protonated nitrogen NBO < -0.45?

Yes (IC_{50} > 7 μM) No (go to Q3)

Clozapine-*N*-oxide

Q3: Is the protonated nitrogen Mulliken > -0.90?

Yes (IC_{50} > 10 μM) No (go to Q4)

Nicardipine

Q4: Is there a carboxylic acid in the molecule?

Yes (IC_{50} > 4 μM) No (go to Q5)

Bilastine
Carebastine
2-Carboxy-methyl-olanzapine
Levofloxacin
Sparfloxacin

Q5: Does the protonated N experience a field effect (shielding) ?

Yes (IC_{50} > 4 μM) No (go to Q6)

Rupatadine

Q6: Does the drug contain zero, one, two, three or more aromatic rings?

One	Two	Three	Four
DSP-6952 (0.271)	Azimilide (0.58)	Clozapine (0.32)	O-DesMe astemizole (0.01)
Mexiletine (>10)	CJ-033466 (2.6)	Desmethyl clozapine (4.49)	
	Chlorphenamine (13)	Desmethyl-olanzapine (14.2)	
	Dofetilide (0.007)	Ebastine (0.30)	
	Dolasetron (5.95)	Endoxifen (1.6)	
	E-4031 (0.0268)	2-OH-methyl-olanzapine (0.23)	
	MDL 74,156 (12.1)	9-OH-Risperidone (1.3)	
	MK-499 (0.032)	Lidoflazine (0.037)	
	Propafenone (2.0)	Mibefradil (1.43)	
	Ave = 4.5 μM	Norastemizole (0.028)	
		Olanzapine (0.231)	
		Prenylamine (0.59)	
		Risperidone (0.14)	
		Sertindole (0.024)	
		Sildenafil (3.3)	
		Ziprasidone (0.15)	
		Ave = 1.8 μM	

Q7: For drugs with more than one aromatic ring, are they going in the same direction or are they going in different directions? What is the number of atoms away from the protonated amine? See **Table S4**

Table S4 Summary of Q6 for compound test set

IC50 μM	Two Aromatic Rings	N, Alkyl	Distance from amine	Direction
0.007	Dofetilide	Tertiary; Me-Et-2-ethoxy (102 amu)	2 and 3 BDS, Sep by 6 BDS	Opposite
0.0268	E-4031	Tertiary; Et-4-CO-piperazine (111 amu)	2 and 4 BDS, Sep by 7 BDS	Opposite
0.032	MK-499	Tert; 4-Disub-piperaine-cyclohex (138 amu)	2 and 4 BDS, Sep by 7 BDS	Opposite
0.58	Azimilide	Tertiary; ethyl-4-CO-piperazine (152 amu)	11 and 14 BDS, Sep by 0 BDS	Same
2.00	Propafenone	Secondary; propyl-2-OH-3O-propyl (133 amu)	4 and 9 BDS, Sep by 3 BDS	Same
2.60	CJ-033466	Tert;N-isopro-piper-CH_2NH-CO-R (155 amu)	6 and 7 BDS, Sep by 0 BDS	Same
Est 3-4	Sumatriptan	Tertiary; dimethyl-ethyl (73 amu)	2 and 3 BDS, Sep by 0 BDS	Same
5.95	Dolasetron	Tertiary; adamantanone (165 amu)	4 and 6 BDS, Sep by 0 BDS	Same
12.1	MDL 74,156	Tertiary; -2-OH-adamantane (155 amu)	4 and 6 BDS, Sep by 1 bond	Same
13.0	Chlorphenamine	Tertiary; dimethyl-propyl (86 amu)	3 and 3 BDS, Sep by 1 BDS	Same

IC50 μM	3 Aromatic Rings	N Alkyl	Distance from amine	Direction
0.02	Sertindole	Tert; 2-cyclopentyl-4-piperidine (126 amu)	3, 4 and 6 BDS, Sep 0 BDS	Same
0.028	Norastemizole	Secondary; 4-NHR-piperazine (100 amu)	4, 6, and 7 BDS, Sep 1	Same
0.037	Lidoflazine	Tert; 4-N-R-piperidine-1-butyl (154 amu)	4,4 and 6 BDS, Sep 11 BDS	Opposite
0.14	Risperidone	Tertiary; 2-R-Et-4-R'-piperidine (112 amu)	2,3,4 BDS, Sep 6	Opposite
0.15	Ziprasidone	Tertiary; 2-R-Et-4-R'piperazine (112 amu)	2, 3, and 4 BDS, Sep 6 BDS	Opposite
0.23	2-OH-Me-olanzapine	Tertiary; Me-4-R-piperazine (100 amu)	3, 4 and 5 BDS, Sep 0,1 BDS	Same
0.231	Olanzapine	Tertiary; Me-4-R-piperazine (100 amu)	3, 4, and 5 BDS, Sep 0 BDS	Same
0.30	Ebastine	Tert; 4-oxo-but-(4-OCHRR')-pip (142 amu)	4, 5, and 5 BDS, Sep 10 BDS	Opposite
0.59	Prenylamine	2ndary; 3,3-RR'-prop-isoprop-2-R (99 amu)	2, 3, and 3 BDS, Sep 6 BDS	Opposite
1.3	9-OH-Me-risperidone	Tert; 2-R-Et-4-R'-piperidine (112 amu)	2, 3 and 4 BDS, Sep 6 BDS	Opposite
1.43	Mibefradil	Tertiary; methyl-ethyl-3-R-propyl (112 amu)	3, 4 and 5 BDS, Sep 8 BDS	Opposite
1.6	Endoxifen	Secondary; methyl-CH_2OR (75 amu)	3, 8 and 9 BDS, Sep 1 and 2 BDS	Same
3.3	Sildenafil	Tertiary; methyl-4-piperazIne-R (100 amu)	4, 7 and 9 BDS, Sep 0 BDS	Same
4.49	Desmethyl-clozapine	Secondary; piperazine (86 amu)	3, 4 and 5 BDS, Sep 0 BDS	Same
8.1	Rupatadine	Tertiary; 4-R-(N-CH2-R')-piperidine (97 amu)	1, and 4,4 BDS, Sep 6 BDS	Opposite
14.2	Desmethyl-olanzapine	Secondary; 4-R-piperidine (86 amu)	3, 4, and 5 BDS, Sep 0 BDS	Same

IC50 μM	4 aromatic rings	N Alkyl	atom distance from amine	Direction
0.01	O-DesMe-Astemizole	Tertiary;	2-Et-R-(4-NHR'-piperazine) (127 amu)	2, 4, 6 and 7, Sep by 7 and 10 BDS
